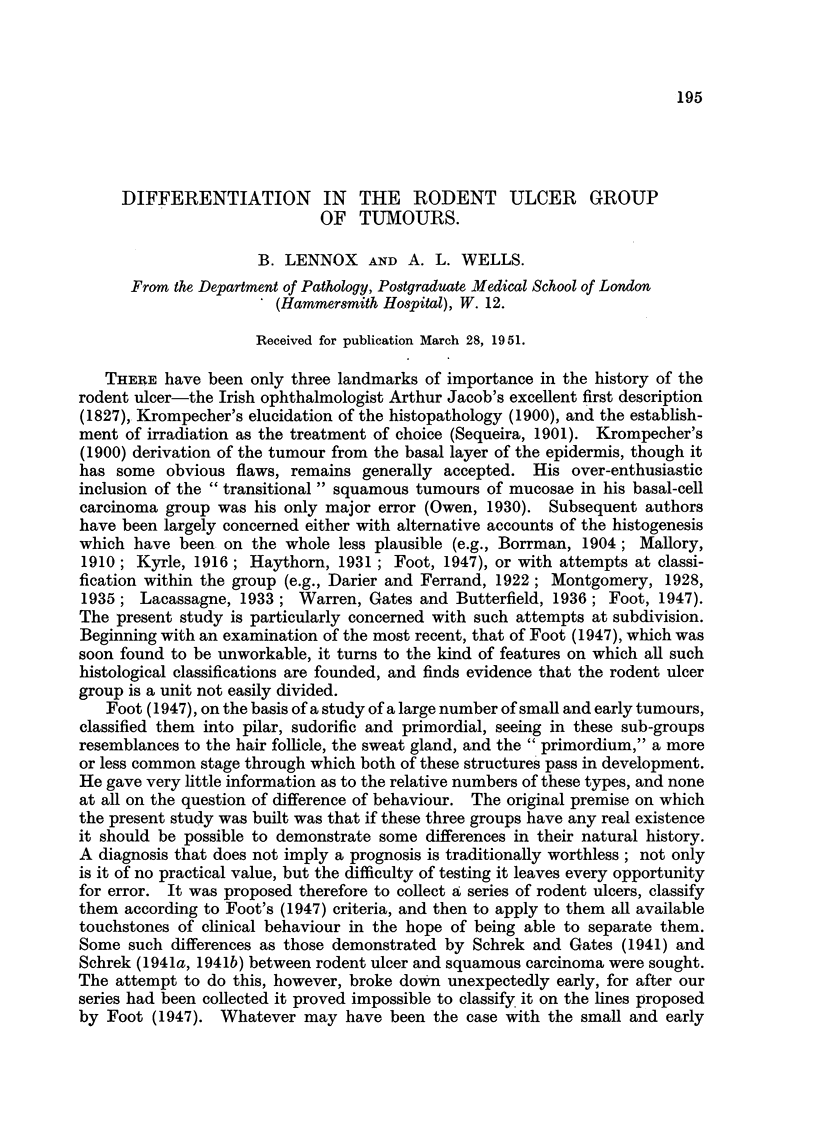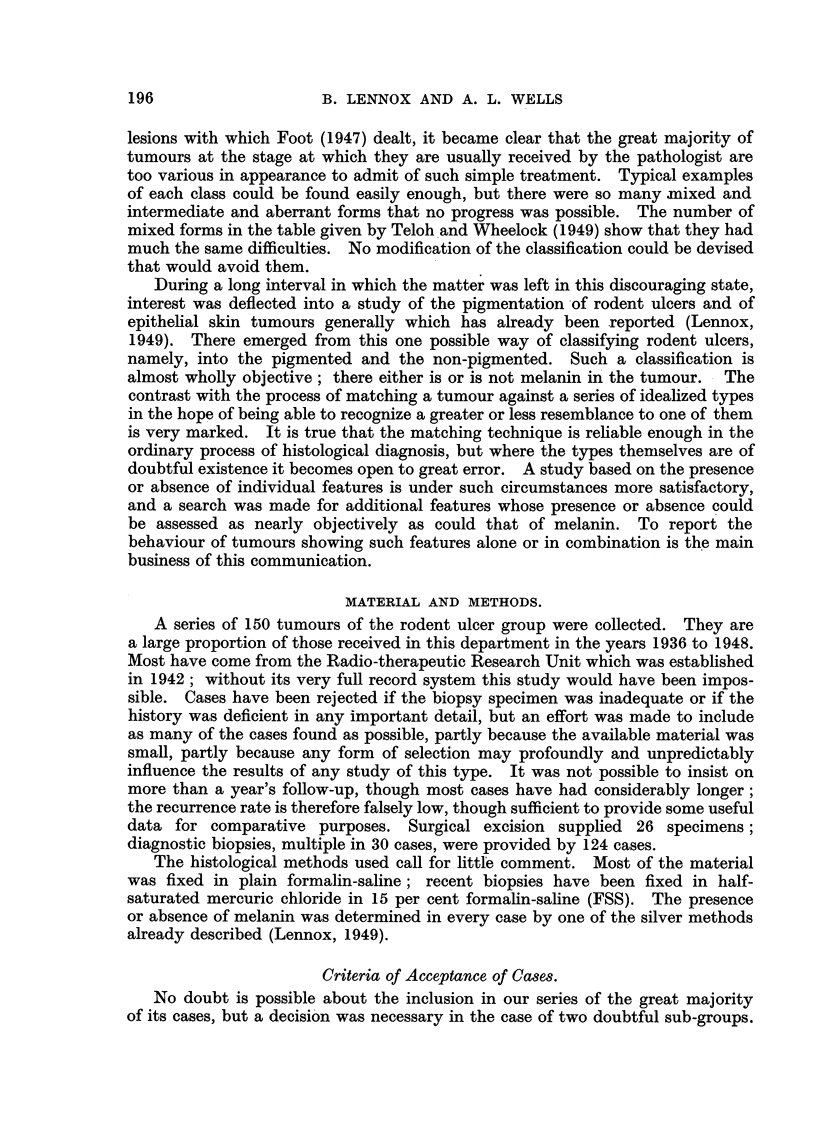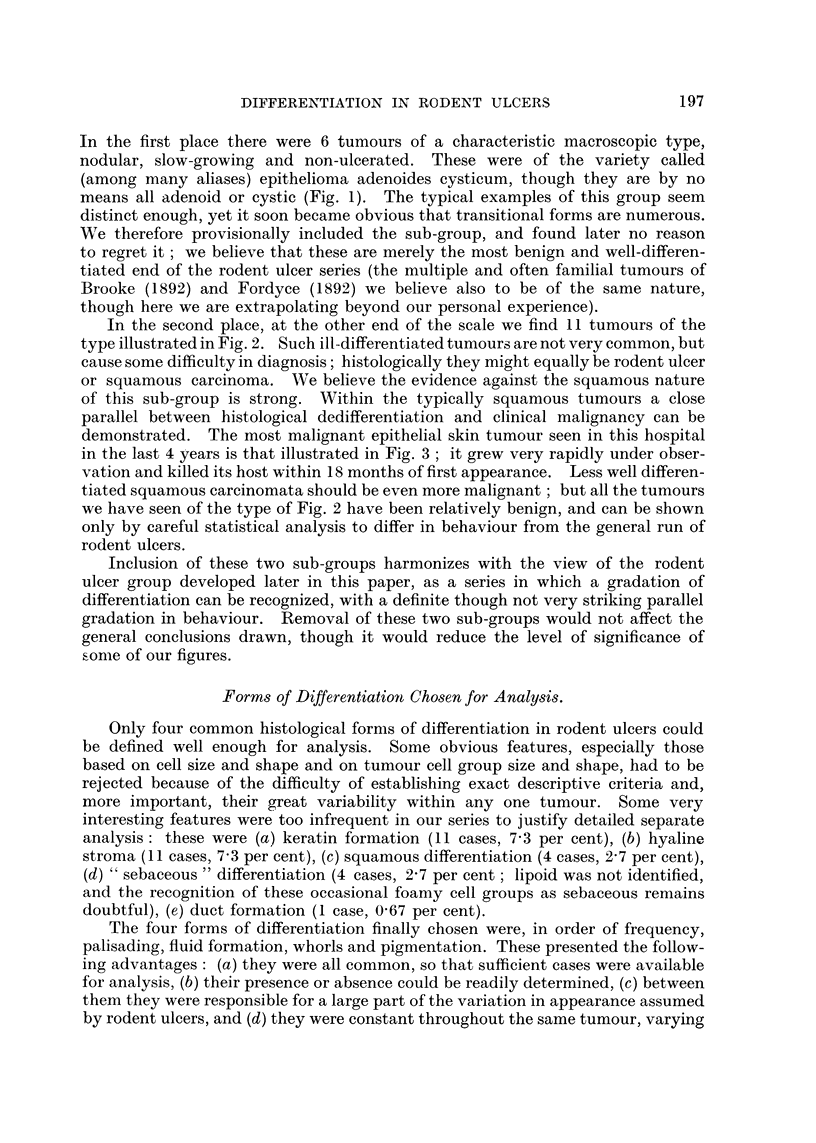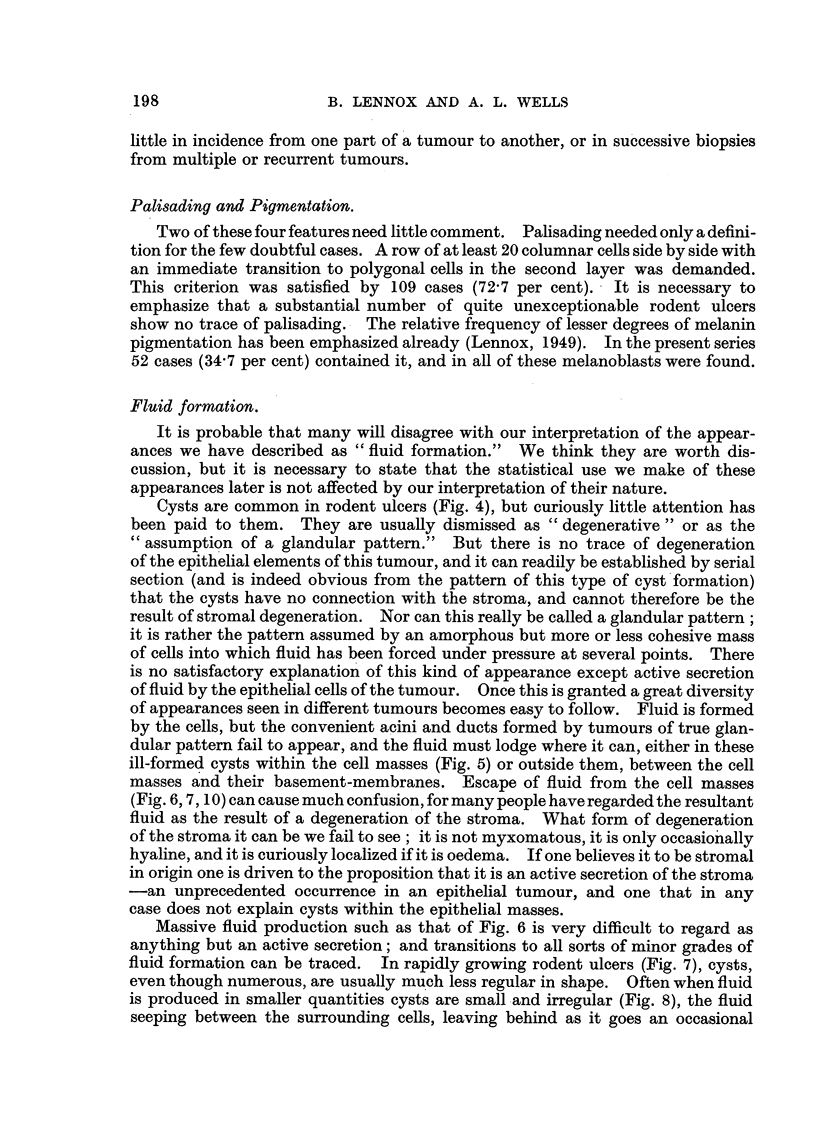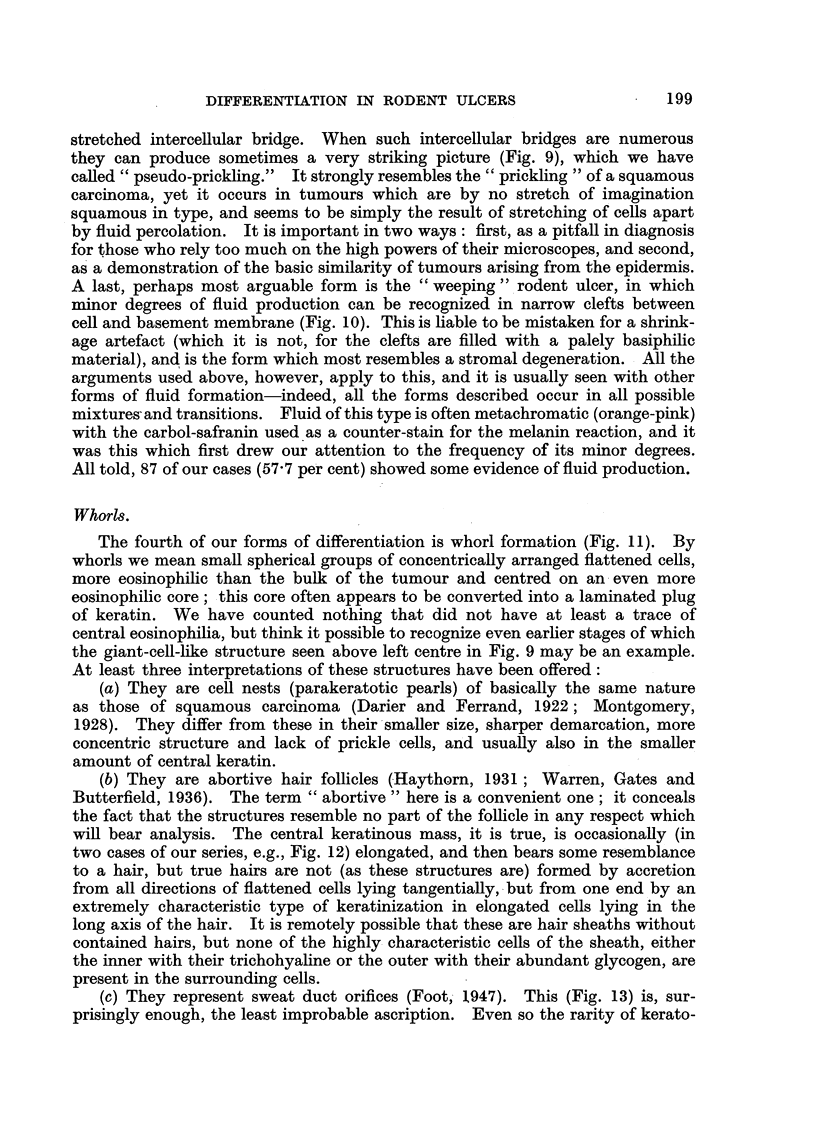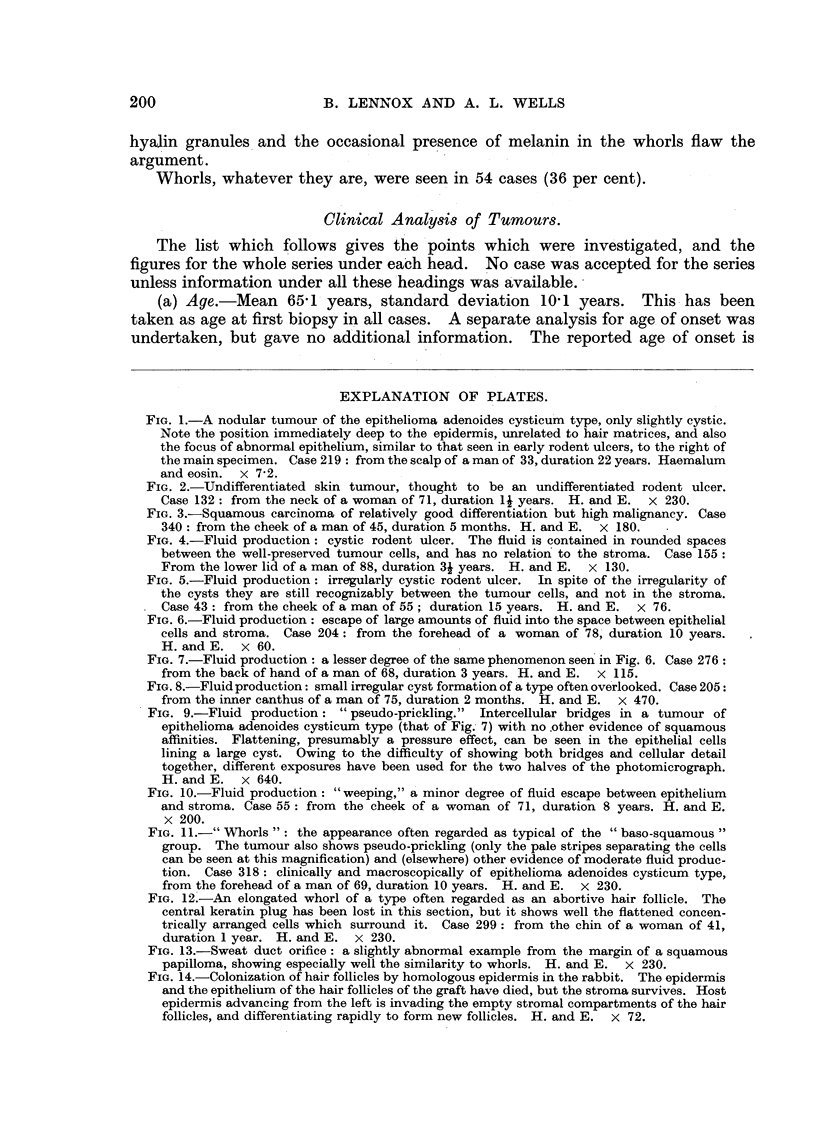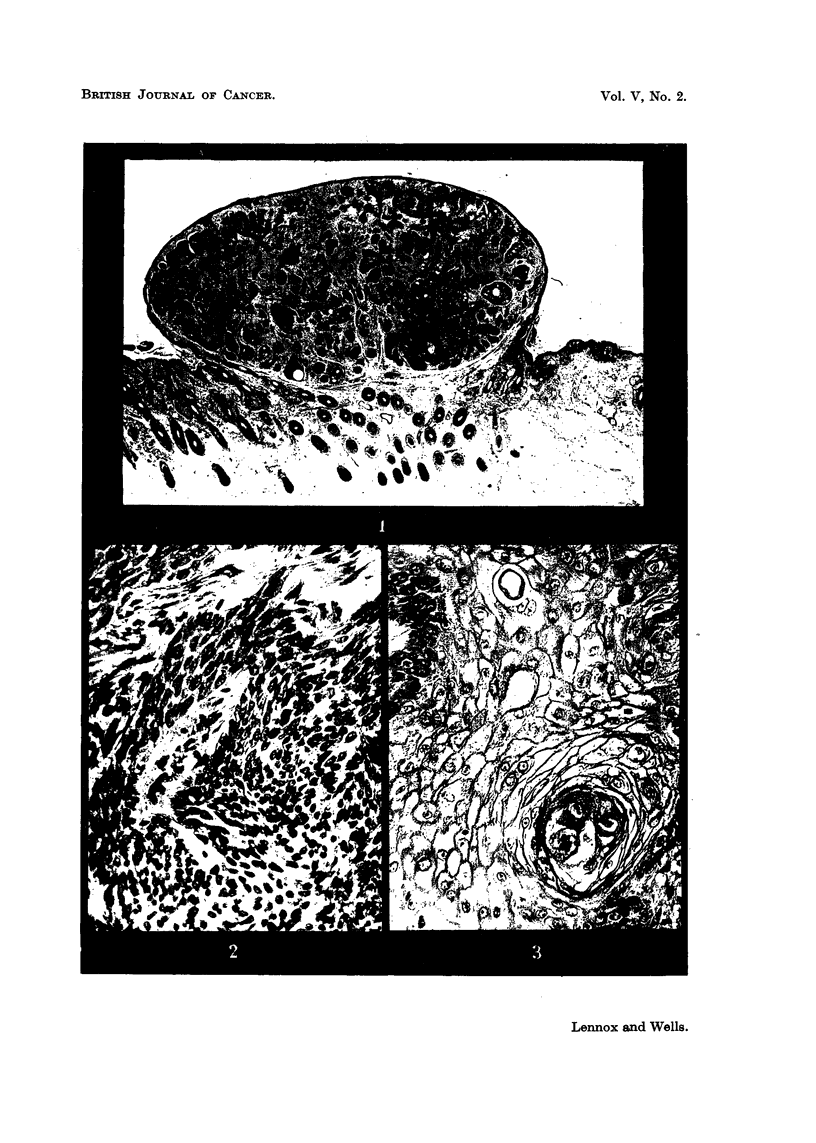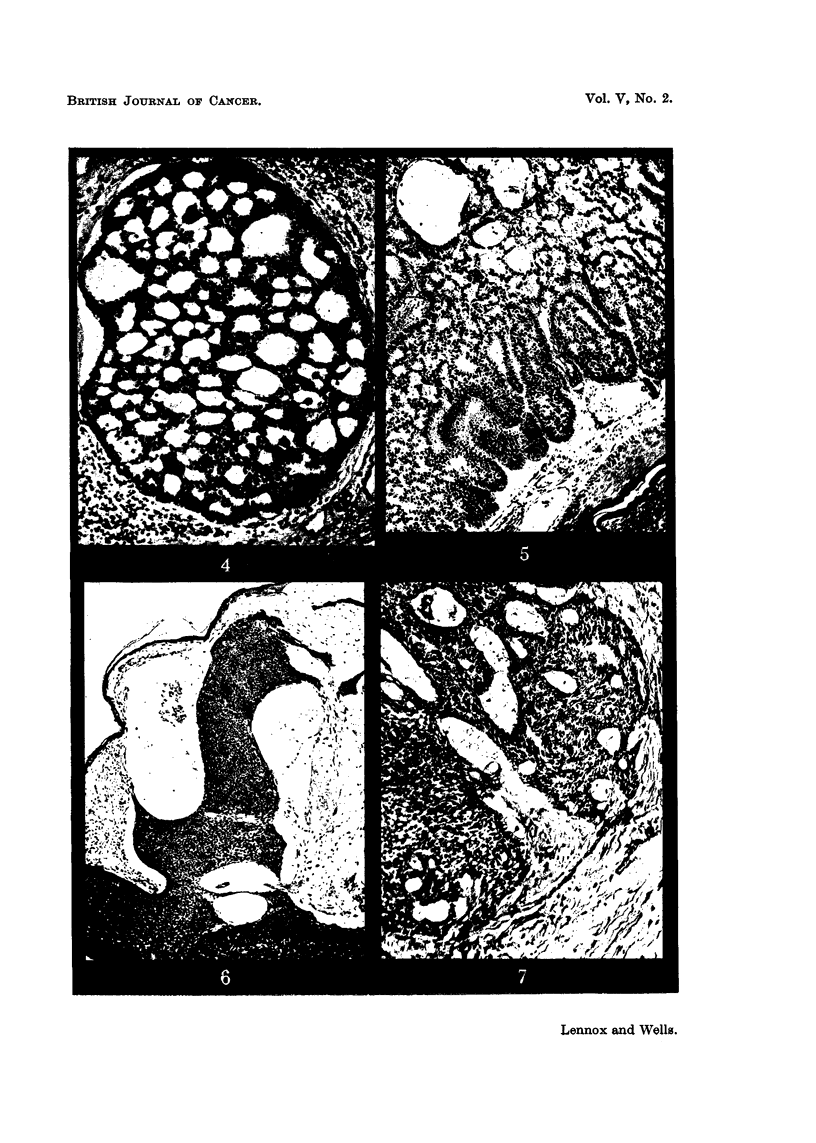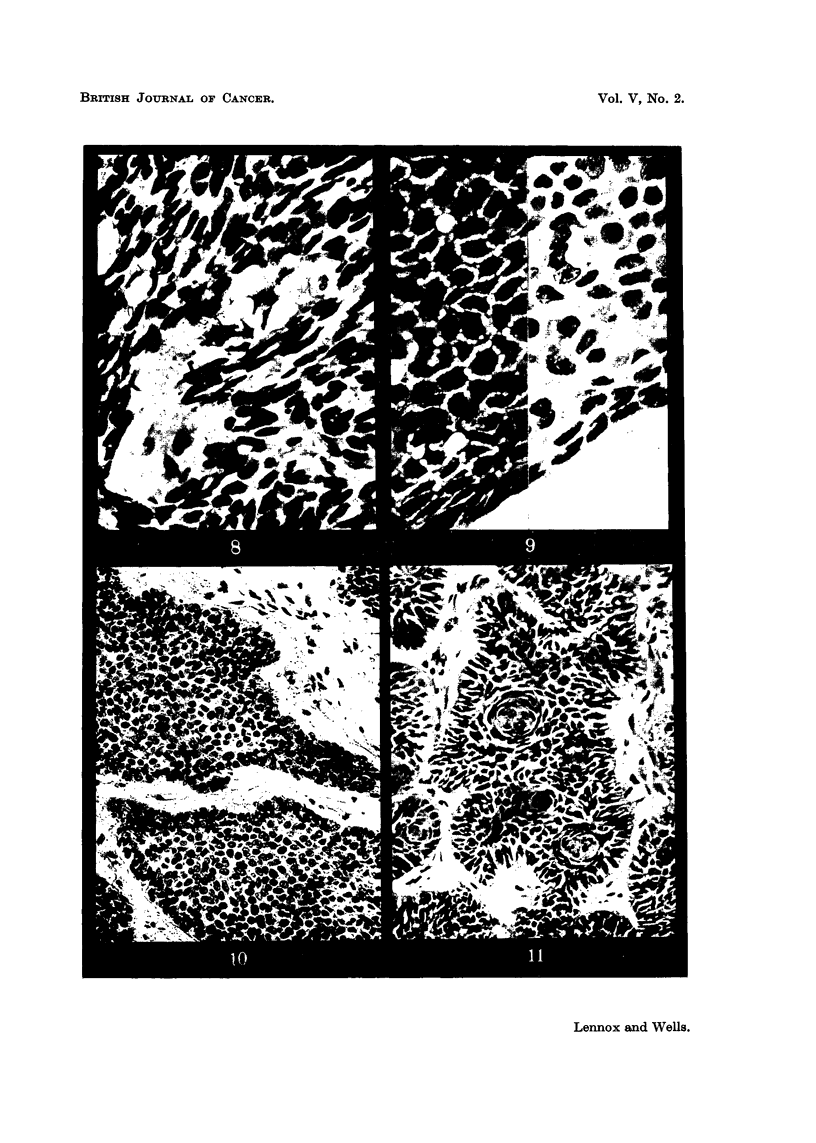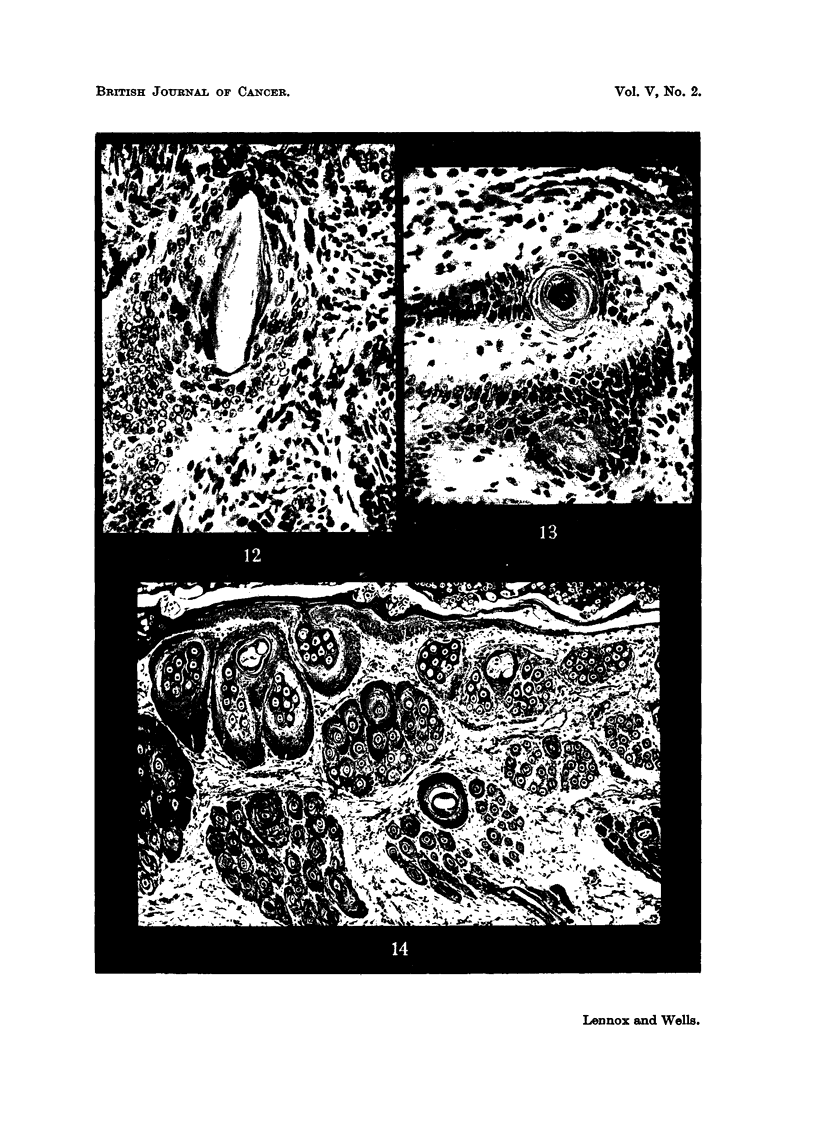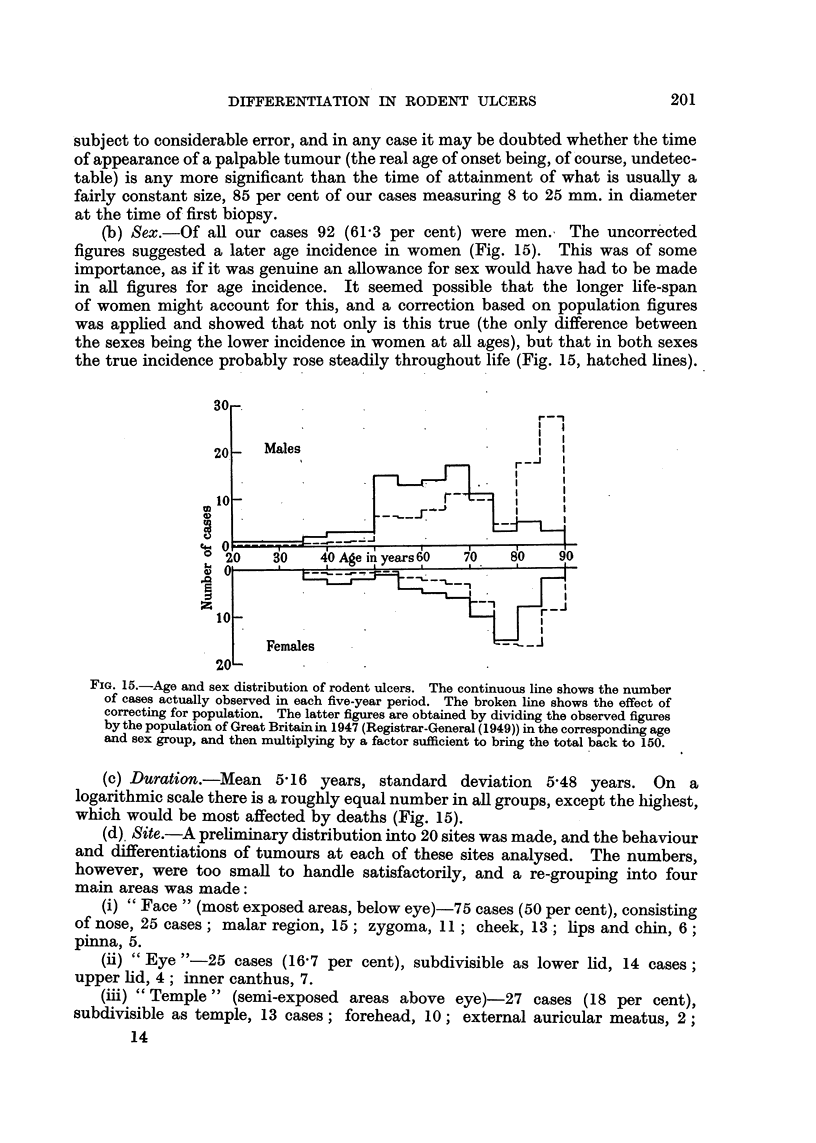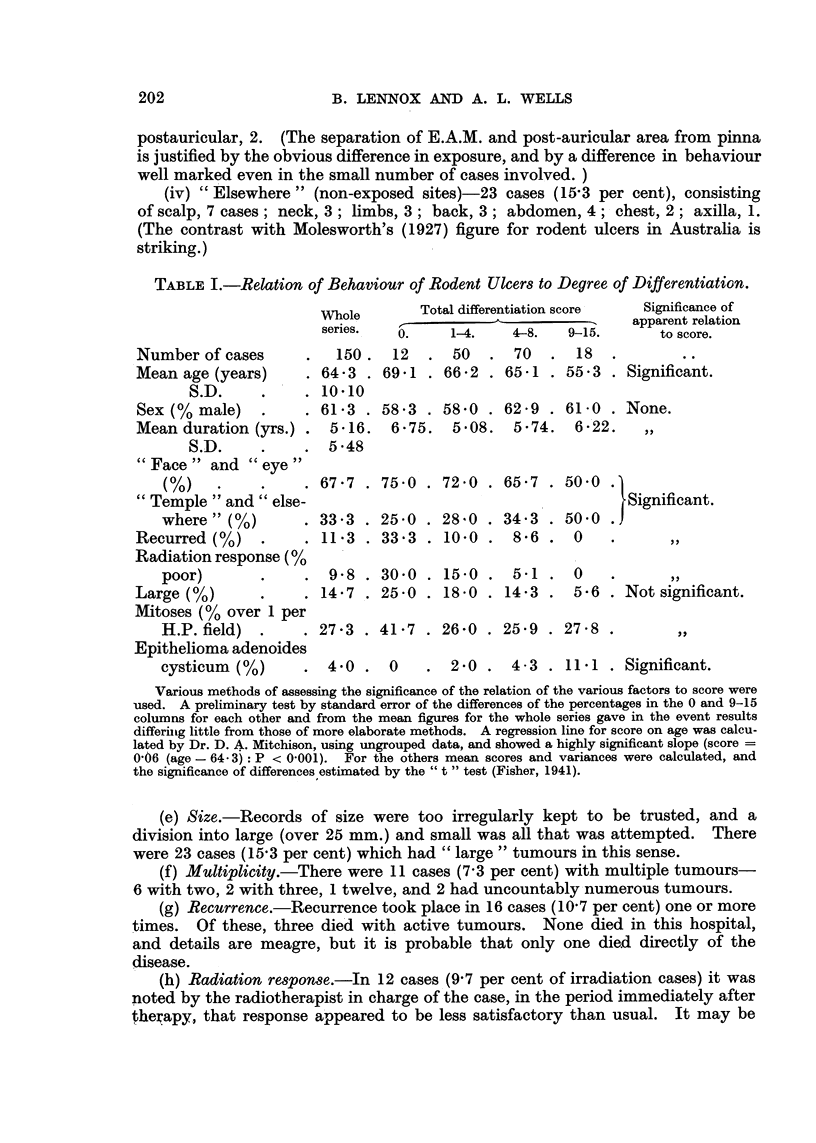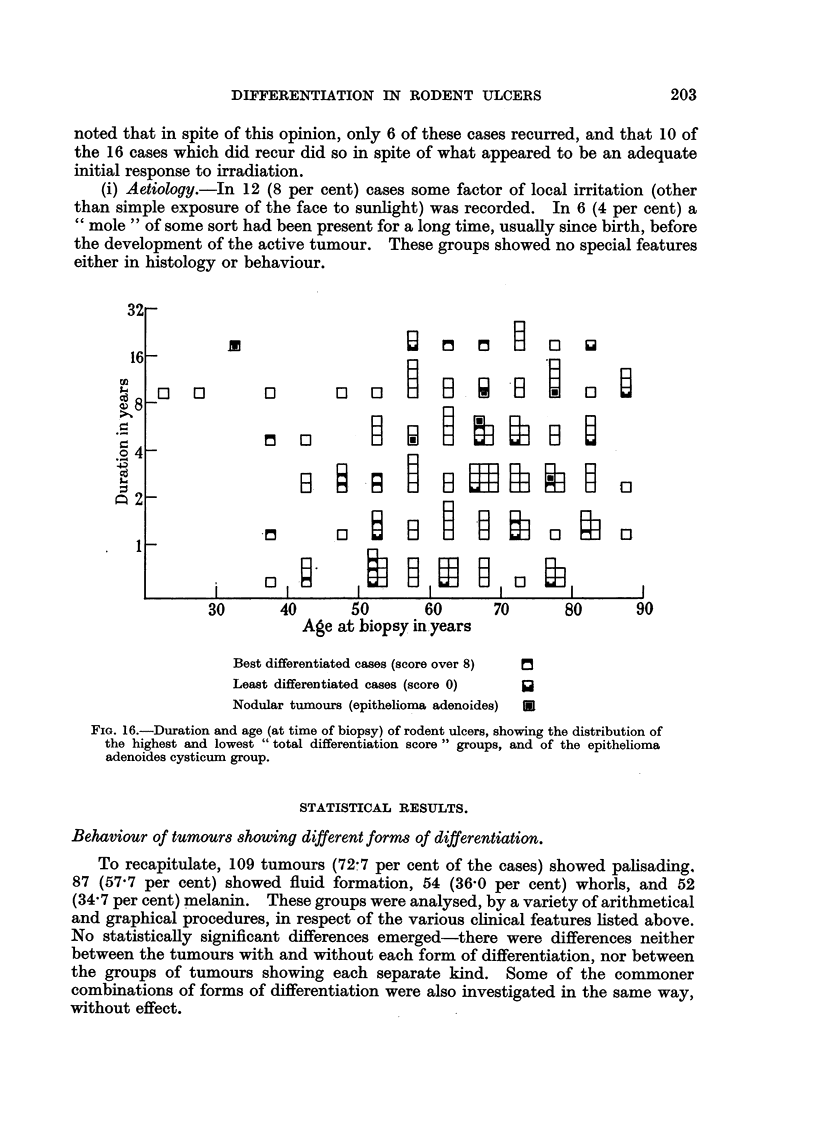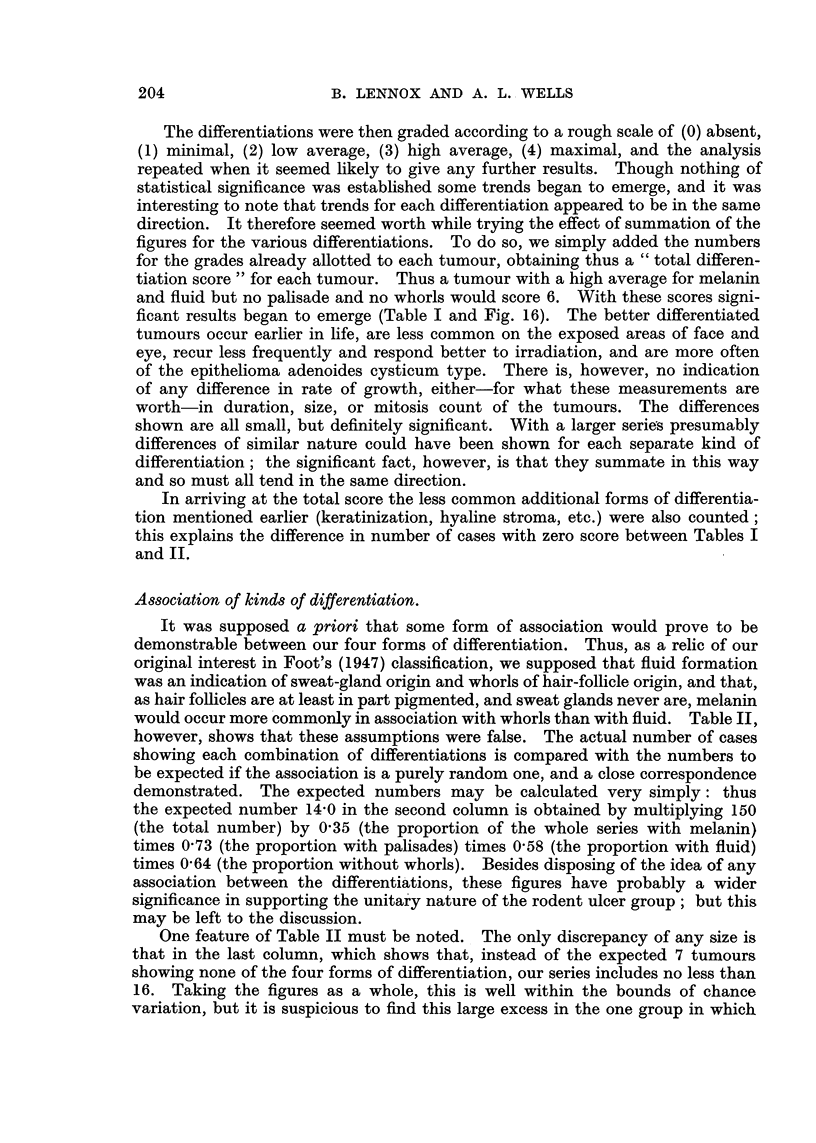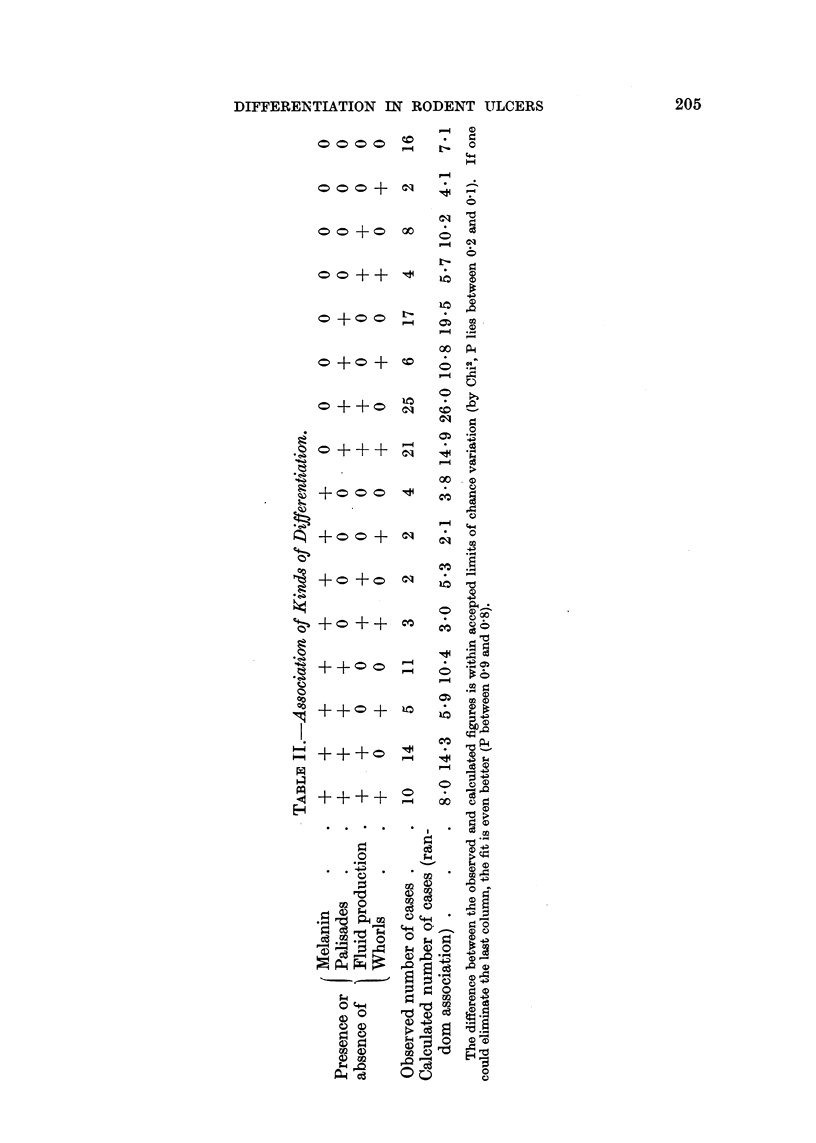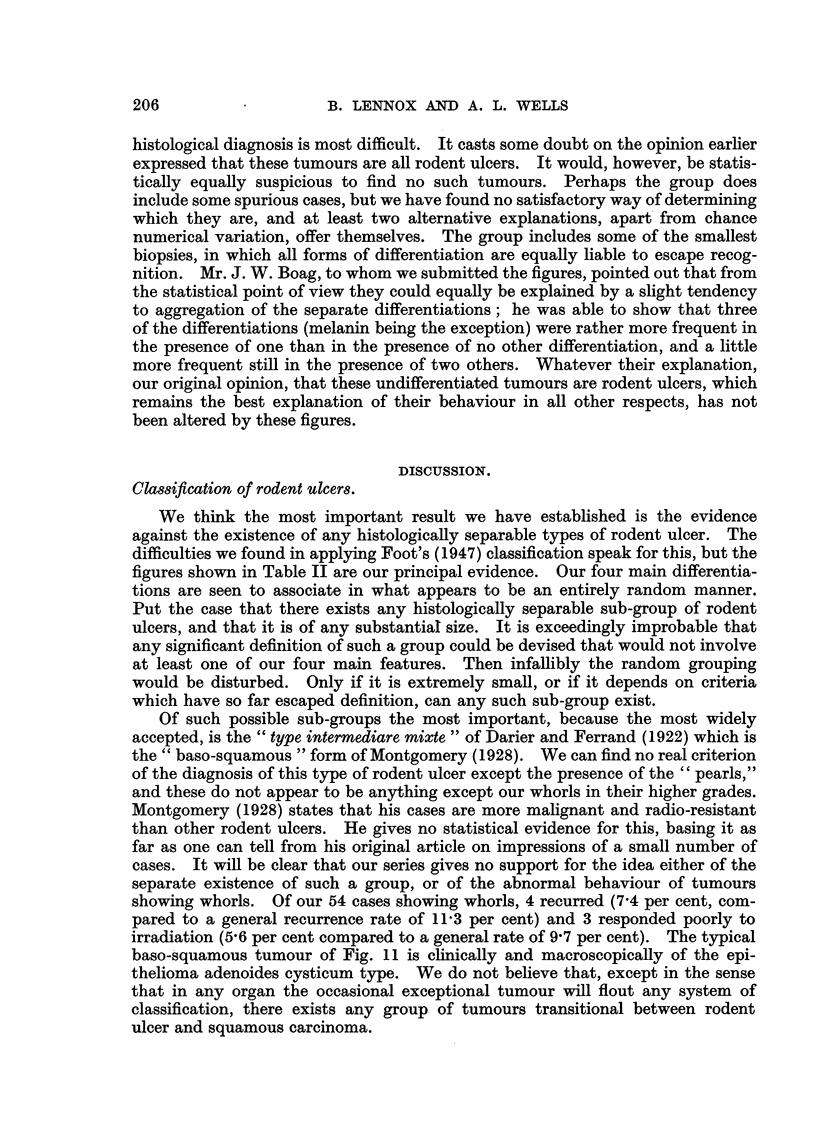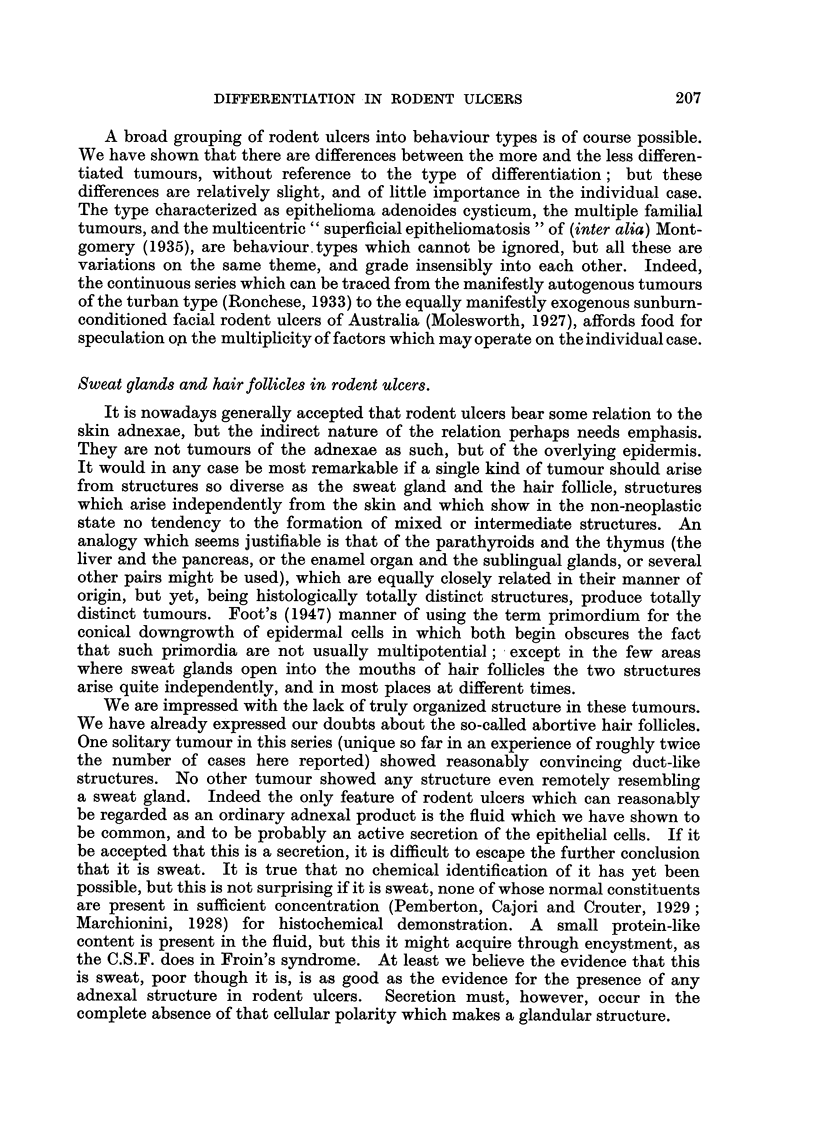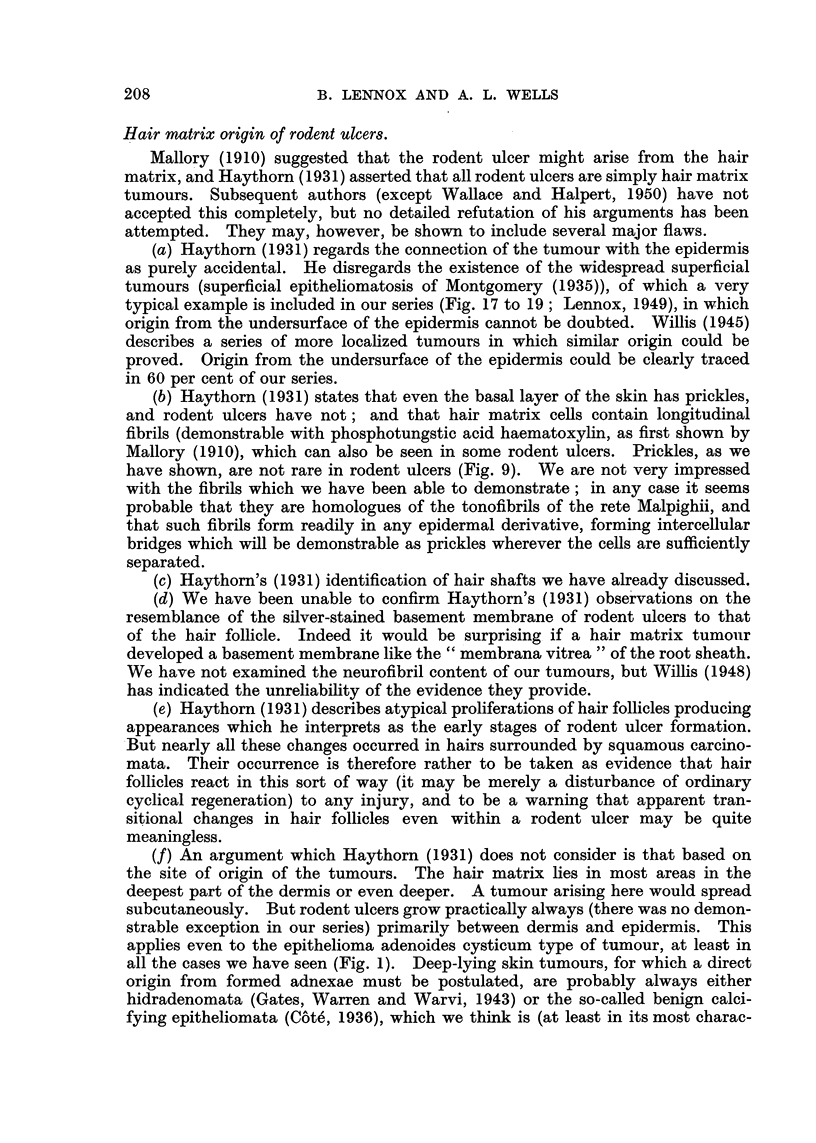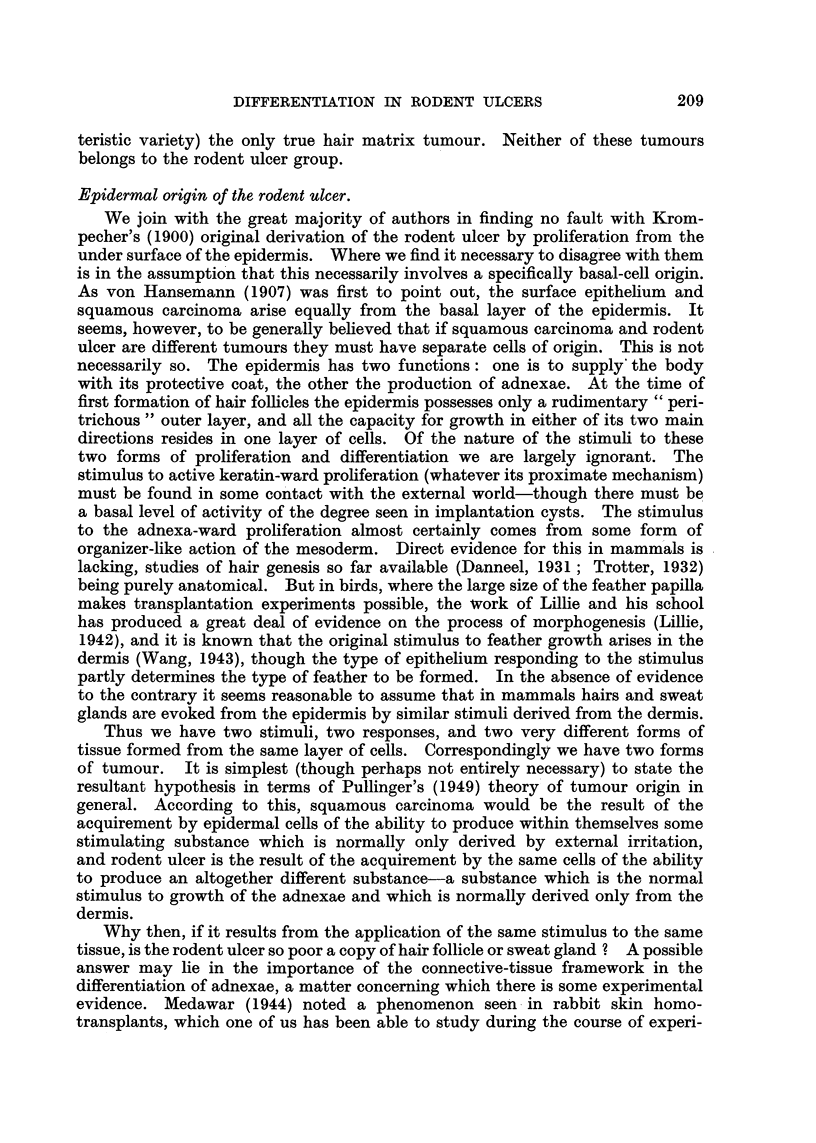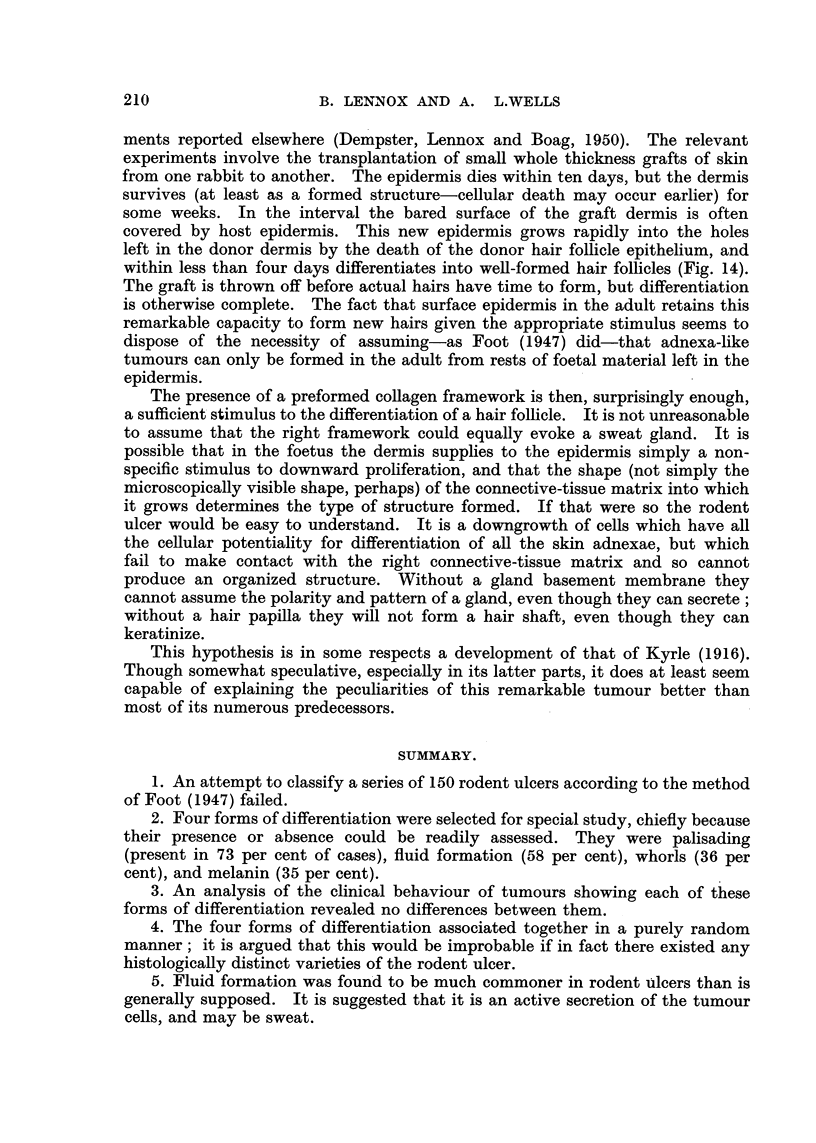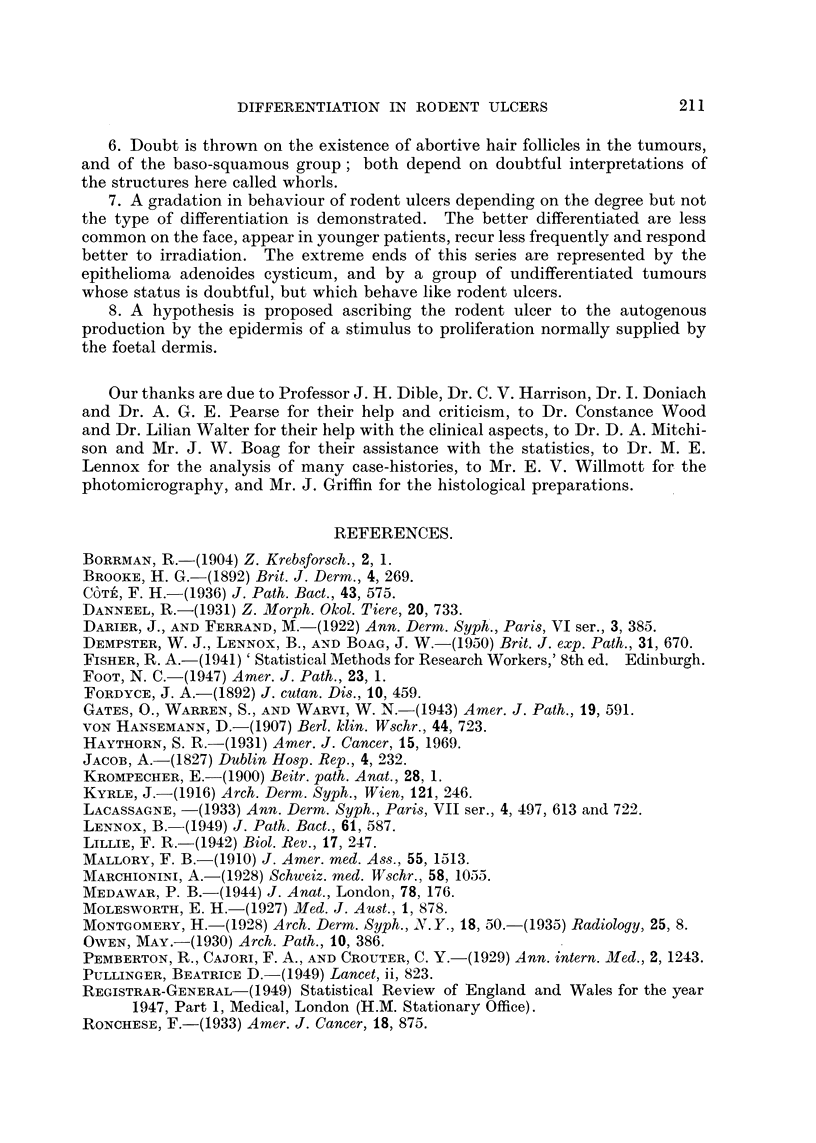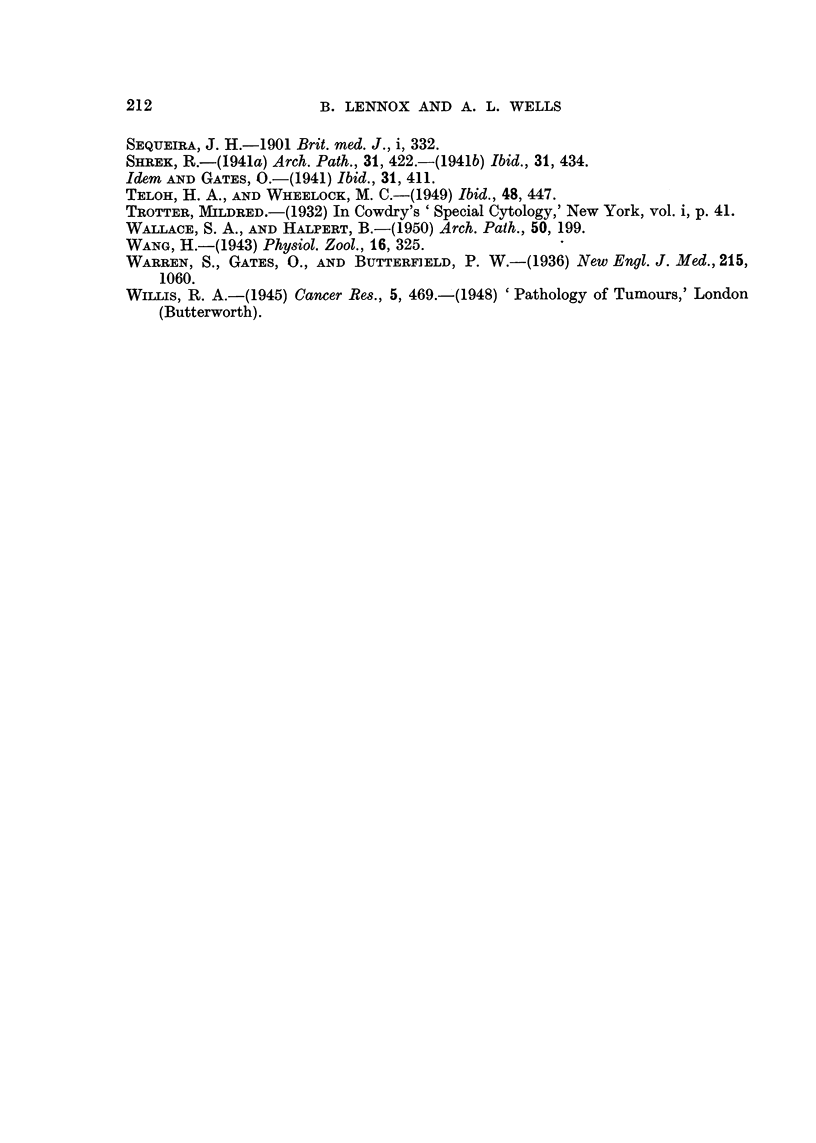# Differentiation in the Rodent Ulcer Group of Tumours

**DOI:** 10.1038/bjc.1951.21

**Published:** 1951-06

**Authors:** B. Lennox, A. L. Wells

## Abstract

**Images:**


### DIFFERENTIATION IN THE RODENT ULCER GROUP OF TUMOURS.

B. LENNOX AND A. L. WELLS.

*From the Department of Pathology, Postgraduate Medical School of London (Hammersmith Hospital), W. 12.*

Received for publication March 28, 1951.

THERE have been only three landmarks of importance in the history of the rodent ulcer—the Irish ophthalmologist Arthur Jacob's excellent first description (1827), Krompecher's elucidation of the histopathology (1900), and the establishment of irradiation as the treatment of choice (Sequeira, 1901). Krompecher's (1900) derivation of the tumour from the basal layer of the epidermis, though it has some obvious flaws, remains generally accepted. His over-enthusiastic inclusion of the "transitional" squamous tumours of mucosae in his basal-cell carcinoma group was his only major error (Owen, 1930). Subsequent authors have been largely concerned either with alternative accounts of the histogenesis which have been on the whole less plausible (e.g., Borrman, 1904; Mallory, 1910; Kyrle, 1916; Haythorn, 1931; Foot, 1947), or with attempts at classification within the group (e.g., Darier and Ferrand, 1922; Montgomery, 1928, 1935; Lacassagne, 1933; Warren, Gates and Butterfield, 1936; Foot, 1947). The present study is particularly concerned with such attempts at subdivision. Beginning with an examination of the most recent, that of Foot (1947), which was soon found to be unworkable, it turns to the kind of features on which all such histological classifications are founded, and finds evidence that the rodent ulcer group is a unit not easily divided.

Foot (1947), on the basis of a study of a large number of small and early tumours, classified them into pilar, sudorific and primordial, seeing in these sub-groups resemblances to the hair follicle, the sweat gland, and the "primordium," a more or less common stage through which both of these structures pass in development. He gave very little information as to the relative numbers of these types, and none at all on the question of difference of behaviour. The original premise on which the present study was built was that if these three groups have any real existence it should be possible to demonstrate some differences in their natural history. A diagnosis that does not imply a prognosis is traditionally worthless; not only is it of no practical value, but the difficulty of testing it leaves every opportunity for error. It was proposed therefore to collect a series of rodent ulcers, classify them according to Foot's (1947) criteria, and then to apply to them all available touchstones of clinical behaviour in the hope of being able to separate them. Some such differences as those demonstrated by Schrek and Gates (1941) and Schrek (1941*a*, 1941*b*) between rodent ulcer and squamous carcinoma were sought. The attempt to do this, however, broke down unexpectedly early, for after our series had been collected it proved impossible to classify it on the lines proposed by Foot (1947). Whatever may have been the case with the small and early lesions with which Foot (1947) dealt, it became clear that the great majority of tumours at the stage at which they are usually received by the pathologist are too various in appearance to admit of such simple treatment. Typical examples of each class could be found easily enough, but there were so many mixed and intermediate and aberrant forms that no progress was possible. The number of mixed forms in the table given by Teloh and Wheelock (1949) show that they had much the same difficulties. No modification of the classification could be devised that would avoid them.

During a long interval in which the matter was left in this discouraging state, interest was deflected into a study of the pigmentation of rodent ulcers and of epithelial skin tumours generally which has already been reported (Lennox, 1949). There emerged from this one possible way of classifying rodent ulcers, namely, into the pigmented and the non-pigmented. Such a classification is almost wholly objective ; there either is or is not melanin in the tumour. The contrast with the process of matching a tumour against a series of idealized types in the hope of being able to recognize a greater or less resemblance to one of them is very marked. It is true that the matching technique is reliable enough in the ordinary process of histological diagnosis, but where the types themselves are of doubtful existence it becomes open to great error. A study based on the presence or absence of individual features is under such circumstances more satisfactory, and a search was made for additional features whose presence or absence could be assessed as nearly objectively as could that of melanin. To report the behaviour of tumours showing such features alone or in combination is the main business of this communication.

### MATERIAL AND METHODS.

A series of 150 tumours of the rodent ulcer group were collected. They are a large proportion of those received in this department in the years 1936 to 1948. Most have come from the Radio-therapeutic Research Unit which was established in 1942 ; without its very full record system this study would have been impossible. Cases have been rejected if the biopsy specimen was inadequate or if the history was deficient in any important detail, but an effort was made to include as many of the cases found as possible, partly because the available material was small, partly because any form of selection may profoundly and unpredictably influence the results of any study of this type. It was not possible to insist on more than a year's follow-up, though most cases have had considerably longer ; the recurrence rate is therefore falsely low, though sufficient to provide some useful data for comparative purposes. Surgical excision supplied 26 specimens ; diagnostic biopsies, multiple in 30 cases, were provided by 124 cases.

The histological methods used call for little comment. Most of the material was fixed in plain formalin-saline ; recent biopsies have been fixed in half-saturated mercuric chloride in 15 per cent formalin-saline (FSS). The presence or absence of melanin was determined in every case by one of the silver methods already described (Lennox, 1949).

### *Criteria of Acceptance of Cases.*

No doubt is possible about the inclusion in our series of the great majority of its cases, but a decision was necessary in the case of two doubtful sub-groups.

In the first place there were 6 tumours of a characteristic macroscopic type, nodular, slow-growing and non-ulcerated. These were of the variety called (among many aliases) epithelioma adenoides cysticum, though they are by no means all adenoid or cystic (Fig. 1). The typical examples of this group seem distinct enough, yet it soon became obvious that transitional forms are numerous. We therefore provisionally included the sub-group, and found later no reason to regret it ; we believe that these are merely the most benign and well-differentiated end of the rodent ulcer series (the multiple and often familial tumours of Brooke (1892) and Fordyce (1892) we believe also to be of the same nature, though here we are extrapolating beyond our personal experience).

In the second place, at the other end of the scale we find 11 tumours of the type illustrated in Fig. 2. Such ill-differentiated tumours are not very common, but cause some difficulty in diagnosis ; histologically they might equally be rodent ulcer or squamous carcinoma. We believe the evidence against the squamous nature of this sub-group is strong. Within the typically squamous tumours a close parallel between histological dedifferentiation and clinical malignancy can be demonstrated. The most malignant epithelial skin tumour seen in this hospital in the last 4 years is that illustrated in Fig. 3 ; it grew very rapidly under observation and killed its host within 18 months of first appearance. Less well differentiated squamous carcinomata should be even more malignant ; but all the tumours we have seen of the type of Fig. 2 have been relatively benign, and can be shown only by careful statistical analysis to differ in behaviour from the general run of rodent ulcers.

Inclusion of these two sub-groups harmonizes with the view of the rodent ulcer group developed later in this paper, as a series in which a gradation of differentiation can be recognized, with a definite though not very striking parallel gradation in behaviour. Removal of these two sub-groups would not affect the general conclusions drawn, though it would reduce the level of significance of some of our figures.

*Forms of Differentiation Chosen for Analysis.*

Only four common histological forms of differentiation in rodent ulcers could be defined well enough for analysis. Some obvious features, especially those based on cell size and shape and on tumour cell group size and shape, had to be rejected because of the difficulty of establishing exact descriptive criteria and, more important, their great variability within any one tumour. Some very interesting features were too infrequent in our series to justify detailed separate analysis : these were (*a*) keratin formation (11 cases, 7·3 per cent), (*b*) hyaline stroma (11 cases, 7·3 per cent), (*c*) squamous differentiation (4 cases, 2·7 per cent), (*d*) " sebaceous " differentiation (4 cases, 2·7 per cent ; lipoid was not identified, and the recognition of these occasional foamy cell groups as sebaceous remains doubtful), (*e*) duct formation (1 case, 0·67 per cent).

The four forms of differentiation finally chosen were, in order of frequency, palisading, fluid formation, whorls and pigmentation. These presented the following advantages : (*a*) they were all common, so that sufficient cases were available for analysis, (*b*) their presence or absence could be readily determined, (*c*) between them they were responsible for a large part of the variation in appearance assumed by rodent ulcers, and (*d*) they were constant throughout the same tumour, varying little in incidence from one part of a tumour to another, or in successive biopsies from multiple or recurrent tumours.

*Palisading and Pigmentation.*

Two of these four features need little comment. Palisading needed only a definition for the few doubtful cases. A row of at least 20 columnar cells side by side with an immediate transition to polygonal cells in the second layer was demanded. This criterion was satisfied by 109 cases (72·7 per cent). It is necessary to emphasize that a substantial number of quite unexceptionable rodent ulcers show no trace of palisading. The relative frequency of lesser degrees of melanin pigmentation has been emphasized already (Lennox, 1949). In the present series 52 cases (34·7 per cent) contained it, and in all of these melanoblasts were found.

*Fluid formation.*

It is probable that many will disagree with our interpretation of the appearances we have described as "fluid formation." We think they are worth discussion, but it is necessary to state that the statistical use we make of these appearances later is not affected by our interpretation of their nature.

Cysts are common in rodent ulcers (Fig. 4), but curiously little attention has been paid to them. They are usually dismissed as "degenerative" or as the "assumption of a glandular pattern." But there is no trace of degeneration of the epithelial elements of this tumour, and it can readily be established by serial section (and is indeed obvious from the pattern of this type of cyst formation) that the cysts have no connection with the stroma, and cannot therefore be the result of stromal degeneration. Nor can this really be called a glandular pattern; it is rather the pattern assumed by an amorphous but more or less cohesive mass of cells into which fluid has been forced under pressure at several points. There is no satisfactory explanation of this kind of appearance except active secretion of fluid by the epithelial cells of the tumour. Once this is granted a great diversity of appearances seen in different tumours becomes easy to follow. Fluid is formed by the cells, but the convenient acini and ducts formed by tumours of true glandular pattern fail to appear, and the fluid must lodge where it can, either in these ill-formed cysts within the cell masses (Fig. 5) or outside them, between the cell masses and their basement-membranes. Escape of fluid from the cell masses (Fig. 6, 7, 10) can cause much confusion, for many people have regarded the resultant fluid as the result of a degeneration of the stroma. What form of degeneration of the stroma it can be we fail to see; it is not myxomatous, it is only occasionally hyaline, and it is curiously localized if it is oedema. If one believes it to be stromal in origin one is driven to the proposition that it is an active secretion of the stroma —an unprecedented occurrence in an epithelial tumour, and one that in any case does not explain cysts within the epithelial masses.

Massive fluid production such as that of Fig. 6 is very difficult to regard as anything but an active secretion; and transitions to all sorts of minor grades of fluid formation can be traced. In rapidly growing rodent ulcers (Fig. 7), cysts, even though numerous, are usually much less regular in shape. Often when fluid is produced in smaller quantities cysts are small and irregular (Fig. 8), the fluid seeping between the surrounding cells, leaving behind as it goes an occasional stretched intercellular bridge. When such intercellular bridges are numerous they can produce sometimes a very striking picture (Fig. 9), which we have called " pseudo-prickling." It strongly resembles the " prickling " of a squamous carcinoma, yet it occurs in tumours which are by no stretch of imagination squamous in type, and seems to be simply the result of stretching of cells apart by fluid percolation. It is important in two ways : first, as a pitfall in diagnosis for those who rely too much on the high powers of their microscopes, and second, as a demonstration of the basic similarity of tumours arising from the epidermis. A last, perhaps most arguable form is the " weeping " rodent ulcer, in which minor degrees of fluid production can be recognized in narrow clefts between cell and basement membrane (Fig. 10). This is liable to be mistaken for a shrinkage artefact (which it is not, for the clefts are filled with a palely basiphilic material), and is the form which most resembles a stromal degeneration. All the arguments used above, however, apply to this, and it is usually seen with other forms of fluid formation—indeed, all the forms described occur in all possible mixtures and transitions. Fluid of this type is often metachromatic (orange-pink) with the carbol-safranin used as a counter-stain for the melanin reaction, and it was this which first drew our attention to the frequency of its minor degrees. All told, 87 of our cases (57·7 per cent) showed some evidence of fluid production.

*Whorls.*

The fourth of our forms of differentiation is whorl formation (Fig. 11). By whorls we mean small spherical groups of concentrically arranged flattened cells, more eosinophilic than the bulk of the tumour and centred on an even more eosinophilic core ; this core often appears to be converted into a laminated plug of keratin. We have counted nothing that did not have at least a trace of central eosinophilia, but think it possible to recognize even earlier stages of which the giant-cell-like structure seen above left centre in Fig. 9 may be an example. At least three interpretations of these structures have been offered :

(*a*) They are cell nests (parakeratotic pearls) of basically the same nature as those of squamous carcinoma (Darier and Ferrand, 1922 ; Montgomery, 1928). They differ from these in their smaller size, sharper demarcation, more concentric structure and lack of prickle cells, and usually also in the smaller amount of central keratin.

(*b*) They are abortive hair follicles (Haythorn, 1931 ; Warren, Gates and Butterfield, 1936). The term " abortive " here is a convenient one ; it conceals the fact that the structures resemble no part of the follicle in any respect which will bear analysis. The central keratinous mass, it is true, is occasionally (in two cases of our series, e.g., Fig. 12) elongated, and then bears some resemblance to a hair, but true hairs are not (as these structures are) formed by accretion from all directions of flattened cells lying tangentially, but from one end by an extremely characteristic type of keratinization in elongated cells lying in the long axis of the hair. It is remotely possible that these are hair sheaths without contained hairs, but none of the highly characteristic cells of the sheath, either the inner with their trichohyaline or the outer with their abundant glycogen, are present in the surrounding cells.

(*c*) They represent sweat duct orifices (Foot, 1947). This (Fig. 13) is, surprisingly enough, the least improbable ascription. Even so the rarity of keratohyalin granules and the occasional presence of melanin in the whorls flaw the argument.

Whorls, whatever they are, were seen in 54 cases (36 per cent).

### *Clinical Analysis of Tumours.*

The list which follows gives the points which were investigated, and the figures for the whole series under each head. No case was accepted for the series unless information under all these headings was available.

(a) *Age.*—Mean 65·1 years, standard deviation 10·1 years. This has been taken as age at first biopsy in all cases. A separate analysis for age of onset was undertaken, but gave no additional information. The reported age of onset is

---

EXPLANATION OF PLATES.

FIG. 1.—A nodular tumour of the epithelioma adenoides cysticum type, only slightly cystic. Note the position immediately deep to the epidermis, unrelated to hair matrices, and also the focus of abnormal epithelium, similar to that seen in early rodent ulcers, to the right of the main specimen. Case 219 : from the scalp of a man of 33, duration 22 years. Haemalum and eosin. × 7·2.

FIG. 2.—Undifferentiated skin tumour, thought to be an undifferentiated rodent ulcer. Case 132 : from the neck of a woman of 71, duration 1½ years. H. and E. × 230.

FIG. 3.—Squamous carcinoma of relatively good differentiation but high malignancy. Case 340 : from the cheek of a man of 45, duration 5 months. H. and E. × 180.

FIG. 4.—Fluid production : cystic rodent ulcer. The fluid is contained in rounded spaces between the well-preserved tumour cells, and has no relation to the stroma. Case 155 : From the lower lid of a man of 88, duration 3½ years. H. and E. × 130.

FIG. 5.—Fluid production : irregularly cystic rodent ulcer. In spite of the irregularity of the cysts they are still recognizably between the tumour cells, and not in the stroma. Case 43 : from the cheek of a man of 55 ; duration 15 years. H. and E. × 76.

FIG. 6.—Fluid production : escape of large amounts of fluid into the space between epithelial cells and stroma. Case 204 : from the forehead of a woman of 78, duration 10 years. H. and E. × 60.

FIG. 7.—Fluid production : a lesser degree of the same phenomenon seen in Fig. 6. Case 276 : from the back of hand of a man of 68, duration 3 years. H. and E. × 115.

FIG. 8.—Fluid production : small irregular cyst formation of a type often overlooked. Case 205 : from the inner canthus of a man of 75, duration 2 months. H. and E. × 470.

FIG. 9.—Fluid production : "pseudo-prickling." Intercellular bridges in a tumour of epithelioma adenoides cysticum type (that of Fig. 7) with no other evidence of squamous affinities. Flattening, presumably a pressure effect, can be seen in the epithelial cells lining a large cyst. Owing to the difficulty of showing both bridges and cellular detail together, different exposures have been used for the two halves of the photomicrograph. H. and E. × 640.

FIG. 10.—Fluid production : "weeping," a minor degree of fluid escape between epithelium and stroma. Case 55 : from the cheek of a woman of 71, duration 8 years. H. and E. × 200.

FIG. 11.—" Whorls " : the appearance often regarded as typical of the " baso-squamous " group. The tumour also shows pseudo-prickling (only the pale stripes separating the cells can be seen at this magnification) and (elsewhere) other evidence of moderate fluid production. Case 318 : clinically and macroscopically of epithelioma adenoides cysticum type, from the forehead of a man of 69, duration 10 years. H. and E. × 230.

FIG. 12.—An elongated whorl of a type often regarded as an abortive hair follicle. The central keratin plug has been lost in this section, but it shows well the flattened concentrically arranged cells which surround it. Case 299 : from the chin of a woman of 41, duration 1 year. H. and E. × 230.

FIG. 13.—Sweat duct orifice : a slightly abnormal example from the margin of a squamous papilloma, showing especially well the similarity to whorls. H. and E. × 230.

FIG. 14.—Colonization of hair follicles by homologous epidermis in the rabbit. The epidermis and the epithelium of the hair follicles of the graft have died, but the stroma survives. Host epidermis advancing from the left is invading the empty stromal compartments of the hair follicles, and differentiating rapidly to form new follicles. H. and E. × 72.

1

2

3

4 5 6 7

Lennox and Wells.

8 9 10 11

12

13

14

subject to considerable error, and in any case it may be doubted whether the time of appearance of a palpable tumour (the real age of onset being, of course, undetectable) is any more significant than the time of attainment of what is usually a fairly constant size, 85 per cent of our cases measuring 8 to 25 mm. in diameter at the time of first biopsy.

(b) *Sex.*—Of all our cases 92 (61·3 per cent) were men. The uncorrected figures suggested a later age incidence in women (Fig. 15). This was of some importance, as if it was genuine an allowance for sex would have had to be made in all figures for age incidence. It seemed possible that the longer life-span of women might account for this, and a correction based on population figures was applied and showed that not only is this true (the only difference between the sexes being the lower incidence in women at all ages), but that in both sexes the true incidence probably rose steadily throughout life (Fig. 15, hatched lines).

FIG. 15.—Age and sex distribution of rodent ulcers. The continuous line shows the number of cases actually observed in each five-year period. The broken line shows the effect of correcting for population. The latter figures are obtained by dividing the observed figures by the population of Great Britain in 1947 (Registrar-General (1949)) in the corresponding age and sex group, and then multiplying by a factor sufficient to bring the total back to 150.

(c) *Duration.*—Mean 5·16 years, standard deviation 5·48 years. On a logarithmic scale there is a roughly equal number in all groups, except the highest, which would be most affected by deaths (Fig. 15).

(d) *Site.*—A preliminary distribution into 20 sites was made, and the behaviour and differentiations of tumours at each of these sites analysed. The numbers, however, were too small to handle satisfactorily, and a re-grouping into four main areas was made:

(i) "Face" (most exposed areas, below eye)—75 cases (50 per cent), consisting of nose, 25 cases; malar region, 15; zygoma, 11; cheek, 13; lips and chin, 6; pinna, 5.

(ii) "Eye"—25 cases (16·7 per cent), subdivisible as lower lid, 14 cases; upper lid, 4; inner canthus, 7.

(iii) "Temple" (semi-exposed areas above eye)—27 cases (18 per cent), subdivisible as temple, 13 cases; forehead, 10; external auricular meatus, 2; postauricular, 2. (The separation of E.A.M. and post-auricular area from pinna is justified by the obvious difference in exposure, and by a difference in behaviour well marked even in the small number of cases involved. )

(iv) " Elsewhere " (non-exposed sites)—23 cases (15·3 per cent), consisting of scalp, 7 cases ; neck, 3 ; limbs, 3 ; back, 3 ; abdomen, 4 ; chest, 2 ; axilla, 1. (The contrast with Molesworth's (1927) figure for rodent ulcers in Australia is striking.)

TABLE I.—*Relation of Behaviour of Rodent Ulcers to Degree of Differentiation.*

| | Whole series. | Total differentiation score 0. | 1–4. | 4–8. | 9–15. | Significance of apparent relation to score. |
|---|---|---|---|---|---|---|
| Number of cases | 150 | 12 | 50 | 70 | 18 | .. |
| Mean age (years) | 64·3 | 69·1 | 66·2 | 65·1 | 55·3 | Significant. |
| S.D. | 10·10 | | | | | |
| Sex (% male) | 61·3 | 58·3 | 58·0 | 62·9 | 61·0 | None. |
| Mean duration (yrs.) | 5·16 | 6·75 | 5·08 | 5·74 | 6·22 | ,, |
| S.D. | 5·48 | | | | | |
| " Face " and " eye " (%) | 67·7 | 75·0 | 72·0 | 65·7 | 50·0 | Significant. (both rows) |
| " Temple " and " elsewhere " (%) | 33·3 | 25·0 | 28·0 | 34·3 | 50·0 | |
| Recurred (%) | 11·3 | 33·3 | 10·0 | 8·6 | 0 | ,, |
| Radiation response (% poor) | 9·8 | 30·0 | 15·0 | 5·1 | 0 | ,, |
| Large (%) | 14·7 | 25·0 | 18·0 | 14·3 | 5·6 | Not significant. |
| Mitoses (% over 1 per H.P. field) | 27·3 | 41·7 | 26·0 | 25·9 | 27·8 | ,, |
| Epithelioma adenoides cysticum (%) | 4·0 | 0 | 2·0 | 4·3 | 11·1 | Significant. |

Various methods of assessing the significance of the relation of the various factors to score were used. A preliminary test by standard error of the differences of the percentages in the 0 and 9–15 columns for each other and from the mean figures for the whole series gave in the event results differing little from those of more elaborate methods. A regression line for score on age was calculated by Dr. D. A. Mitchison, using ungrouped data, and showed a highly significant slope (score = 0·06 (age – 64·3) : P < 0·001). For the others mean scores and variances were calculated, and the significance of differences estimated by the " t " test (Fisher, 1941).

(e) *Size.*—Records of size were too irregularly kept to be trusted, and a division into large (over 25 mm.) and small was all that was attempted. There were 23 cases (15·3 per cent) which had " large " tumours in this sense.

(f) *Multiplicity.*—There were 11 cases (7·3 per cent) with multiple tumours—6 with two, 2 with three, 1 twelve, and 2 had uncountably numerous tumours.

(g) *Recurrence.*—Recurrence took place in 16 cases (10·7 per cent) one or more times. Of these, three died with active tumours. None died in this hospital, and details are meagre, but it is probable that only one died directly of the disease.

(h) *Radiation response.*—In 12 cases (9·7 per cent of irradiation cases) it was noted by the radiotherapist in charge of the case, in the period immediately after therapy, that response appeared to be less satisfactory than usual. It may be noted that in spite of this opinion, only 6 of these cases recurred, and that 10 of the 16 cases which did recur did so in spite of what appeared to be an adequate initial response to irradiation.

(i) *Aetiology.*—In 12 (8 per cent) cases some factor of local irritation (other than simple exposure of the face to sunlight) was recorded. In 6 (4 per cent) a " mole " of some sort had been present for a long time, usually since birth, before the development of the active tumour. These groups showed no special features either in histology or behaviour.

Best differentiated cases (score over 8)
Least differentiated cases (score 0)
Nodular tumours (epithelioma adenoides)

FIG. 16.—Duration and age (at time of biopsy) of rodent ulcers, showing the distribution of the highest and lowest " total differentiation score " groups, and of the epithelioma adenoides cysticum group.

STATISTICAL RESULTS.

*Behaviour of tumours showing different forms of differentiation.*

To recapitulate, 109 tumours (72·7 per cent of the cases) showed palisading, 87 (57·7 per cent) showed fluid formation, 54 (36·0 per cent) whorls, and 52 (34·7 per cent) melanin. These groups were analysed, by a variety of arithmetical and graphical procedures, in respect of the various clinical features listed above. No statistically significant differences emerged—there were differences neither between the tumours with and without each form of differentiation, nor between the groups of tumours showing each separate kind. Some of the commoner combinations of forms of differentiation were also investigated in the same way, without effect.

The differentiations were then graded according to a rough scale of (0) absent, (1) minimal, (2) low average, (3) high average, (4) maximal, and the analysis repeated when it seemed likely to give any further results. Though nothing of statistical significance was established some trends began to emerge, and it was interesting to note that trends for each differentiation appeared to be in the same direction. It therefore seemed worth while trying the effect of summation of the figures for the various differentiations. To do so, we simply added the numbers for the grades already allotted to each tumour, obtaining thus a " total differentiation score " for each tumour. Thus a tumour with a high average for melanin and fluid but no palisade and no whorls would score 6. With these scores significant results began to emerge (Table I and Fig. 16). The better differentiated tumours occur earlier in life, are less common on the exposed areas of face and eye, recur less frequently and respond better to irradiation, and are more often of the epithelioma adenoides cysticum type. There is, however, no indication of any difference in rate of growth, either—for what these measurements are worth—in duration, size, or mitosis count of the tumours. The differences shown are all small, but definitely significant. With a larger series presumably differences of similar nature could have been shown for each separate kind of differentiation ; the significant fact, however, is that they summate in this way and so must all tend in the same direction.

In arriving at the total score the less common additional forms of differentiation mentioned earlier (keratinization, hyaline stroma, etc.) were also counted ; this explains the difference in number of cases with zero score between Tables I and II.

*Association of kinds of differentiation.*

It was supposed *a priori* that some form of association would prove to be demonstrable between our four forms of differentiation. Thus, as a relic of our original interest in Foot's (1947) classification, we supposed that fluid formation was an indication of sweat-gland origin and whorls of hair-follicle origin, and that, as hair follicles are at least in part pigmented, and sweat glands never are, melanin would occur more commonly in association with whorls than with fluid. Table II, however, shows that these assumptions were false. The actual number of cases showing each combination of differentiations is compared with the numbers to be expected if the association is a purely random one, and a close correspondence demonstrated. The expected numbers may be calculated very simply : thus the expected number 14·0 in the second column is obtained by multiplying 150 (the total number) by 0·35 (the proportion of the whole series with melanin) times 0·73 (the proportion with palisades) times 0·58 (the proportion with fluid) times 0·64 (the proportion without whorls). Besides disposing of the idea of any association between the differentiations, these figures have probably a wider significance in supporting the unitary nature of the rodent ulcer group ; but this may be left to the discussion.

One feature of Table II must be noted. The only discrepancy of any size is that in the last column, which shows that, instead of the expected 7 tumours showing none of the four forms of differentiation, our series includes no less than 16. Taking the figures as a whole, this is well within the bounds of chance variation, but it is suspicious to find this large excess in the one group in which

TABLE II.—*Association of Kinds of Differentiation.*

| | | | | | | | | | | | | | | | | |
|---|---|---|---|---|---|---|---|---|---|---|---|---|---|---|---|---|
| Presence or absence of: Melanin . . | + | + | + | + | + | + | + | + | 0 | 0 | 0 | 0 | 0 | 0 | 0 | 0 |
| Palisades . . | + | + | + | + | 0 | 0 | 0 | 0 | + | + | + | + | 0 | 0 | 0 | 0 |
| Fluid production . | + | + | 0 | 0 | + | + | 0 | 0 | + | + | 0 | 0 | + | + | 0 | 0 |
| Whorls . . | + | 0 | + | 0 | + | 0 | + | 0 | + | 0 | + | 0 | + | 0 | + | 0 |
| Observed number of cases . . | 10 | 14 | 5 | 11 | 3 | 2 | 2 | 4 | 21 | 25 | 6 | 17 | 4 | 8 | 2 | 16 |
| Calculated number of cases (random association) . . . | 8·0 | 14·3 | 5·9 | 10·4 | 3·0 | 5·3 | 2·1 | 3·8 | 14·9 | 26·0 | 10·8 | 19·5 | 5·7 | 10·2 | 4·1 | 7·1 |

The difference between the observed and calculated figures is within accepted limits of chance variation (by Chi$^2$, P lies between 0·2 and 0·1). If one could eliminate the last column, the fit is even better (P between 0·9 and 0·8).

histological diagnosis is most difficult. It casts some doubt on the opinion earlier expressed that these tumours are all rodent ulcers. It would, however, be statistically equally suspicious to find no such tumours. Perhaps the group does include some spurious cases, but we have found no satisfactory way of determining which they are, and at least two alternative explanations, apart from chance numerical variation, offer themselves. The group includes some of the smallest biopsies, in which all forms of differentiation are equally liable to escape recognition. Mr. J. W. Boag, to whom we submitted the figures, pointed out that from the statistical point of view they could equally be explained by a slight tendency to aggregation of the separate differentiations ; he was able to show that three of the differentiations (melanin being the exception) were rather more frequent in the presence of one than in the presence of no other differentiation, and a little more frequent still in the presence of two others. Whatever their explanation, our original opinion, that these undifferentiated tumours are rodent ulcers, which remains the best explanation of their behaviour in all other respects, has not been altered by these figures.

### DISCUSSION.

*Classification of rodent ulcers.*

We think the most important result we have established is the evidence against the existence of any histologically separable types of rodent ulcer. The difficulties we found in applying Foot's (1947) classification speak for this, but the figures shown in Table II are our principal evidence. Our four main differentiations are seen to associate in what appears to be an entirely random manner. Put the case that there exists any histologically separable sub-group of rodent ulcers, and that it is of any substantial size. It is exceedingly improbable that any significant definition of such a group could be devised that would not involve at least one of our four main features. Then infallibly the random grouping would be disturbed. Only if it is extremely small, or if it depends on criteria which have so far escaped definition, can any such sub-group exist.

Of such possible sub-groups the most important, because the most widely accepted, is the " *type intermediare mixte* " of Darier and Ferrand (1922) which is the " baso-squamous " form of Montgomery (1928). We can find no real criterion of the diagnosis of this type of rodent ulcer except the presence of the " pearls," and these do not appear to be anything except our whorls in their higher grades. Montgomery (1928) states that his cases are more malignant and radio-resistant than other rodent ulcers. He gives no statistical evidence for this, basing it as far as one can tell from his original article on impressions of a small number of cases. It will be clear that our series gives no support for the idea either of the separate existence of such a group, or of the abnormal behaviour of tumours showing whorls. Of our 54 cases showing whorls, 4 recurred (7·4 per cent, compared to a general recurrence rate of 11·3 per cent) and 3 responded poorly to irradiation (5·6 per cent compared to a general rate of 9·7 per cent). The typical baso-squamous tumour of Fig. 11 is clinically and macroscopically of the epithelioma adenoides cysticum type. We do not believe that, except in the sense that in any organ the occasional exceptional tumour will flout any system of classification, there exists any group of tumours transitional between rodent ulcer and squamous carcinoma.

A broad grouping of rodent ulcers into behaviour types is of course possible. We have shown that there are differences between the more and the less differentiated tumours, without reference to the type of differentiation; but these differences are relatively slight, and of little importance in the individual case. The type characterized as epithelioma adenoides cysticum, the multiple familial tumours, and the multicentric "superficial epitheliomatosis" of (*inter alia*) Montgomery (1935), are behaviour types which cannot be ignored, but all these are variations on the same theme, and grade insensibly into each other. Indeed, the continuous series which can be traced from the manifestly autogenous tumours of the turban type (Ronchese, 1933) to the equally manifestly exogenous sunburn-conditioned facial rodent ulcers of Australia (Molesworth, 1927), affords food for speculation on the multiplicity of factors which may operate on the individual case.

*Sweat glands and hair follicles in rodent ulcers.*

It is nowadays generally accepted that rodent ulcers bear some relation to the skin adnexae, but the indirect nature of the relation perhaps needs emphasis. They are not tumours of the adnexae as such, but of the overlying epidermis. It would in any case be most remarkable if a single kind of tumour should arise from structures so diverse as the sweat gland and the hair follicle, structures which arise independently from the skin and which show in the non-neoplastic state no tendency to the formation of mixed or intermediate structures. An analogy which seems justifiable is that of the parathyroids and the thymus (the liver and the pancreas, or the enamel organ and the sublingual glands, or several other pairs might be used), which are equally closely related in their manner of origin, but yet, being histologically totally distinct structures, produce totally distinct tumours. Foot's (1947) manner of using the term primordium for the conical downgrowth of epidermal cells in which both begin obscures the fact that such primordia are not usually multipotential; except in the few areas where sweat glands open into the mouths of hair follicles the two structures arise quite independently, and in most places at different times.

We are impressed with the lack of truly organized structure in these tumours. We have already expressed our doubts about the so-called abortive hair follicles. One solitary tumour in this series (unique so far in an experience of roughly twice the number of cases here reported) showed reasonably convincing duct-like structures. No other tumour showed any structure even remotely resembling a sweat gland. Indeed the only feature of rodent ulcers which can reasonably be regarded as an ordinary adnexal product is the fluid which we have shown to be common, and to be probably an active secretion of the epithelial cells. If it be accepted that this is a secretion, it is difficult to escape the further conclusion that it is sweat. It is true that no chemical identification of it has yet been possible, but this is not surprising if it is sweat, none of whose normal constituents are present in sufficient concentration (Pemberton, Cajori and Crouter, 1929; Marchionini, 1928) for histochemical demonstration. A small protein-like content is present in the fluid, but this it might acquire through encystment, as the C.S.F. does in Froin's syndrome. At least we believe the evidence that this is sweat, poor though it is, is as good as the evidence for the presence of any adnexal structure in rodent ulcers. Secretion must, however, occur in the complete absence of that cellular polarity which makes a glandular structure.

*Hair matrix origin of rodent ulcers.*

Mallory (1910) suggested that the rodent ulcer might arise from the hair matrix, and Haythorn (1931) asserted that all rodent ulcers are simply hair matrix tumours. Subsequent authors (except Wallace and Halpert, 1950) have not accepted this completely, but no detailed refutation of his arguments has been attempted. They may, however, be shown to include several major flaws.

(*a*) Haythorn (1931) regards the connection of the tumour with the epidermis as purely accidental. He disregards the existence of the widespread superficial tumours (superficial epitheliomatosis of Montgomery (1935)), of which a very typical example is included in our series (Fig. 17 to 19 ; Lennox, 1949), in which origin from the undersurface of the epidermis cannot be doubted. Willis (1945) describes a series of more localized tumours in which similar origin could be proved. Origin from the undersurface of the epidermis could be clearly traced in 60 per cent of our series.

(*b*) Haythorn (1931) states that even the basal layer of the skin has prickles, and rodent ulcers have not ; and that hair matrix cells contain longitudinal fibrils (demonstrable with phosphotungstic acid haematoxylin, as first shown by Mallory (1910), which can also be seen in some rodent ulcers. Prickles, as we have shown, are not rare in rodent ulcers (Fig. 9). We are not very impressed with the fibrils which we have been able to demonstrate ; in any case it seems probable that they are homologues of the tonofibrils of the rete Malpighii, and that such fibrils form readily in any epidermal derivative, forming intercellular bridges which will be demonstrable as prickles wherever the cells are sufficiently separated.

(*c*) Haythorn's (1931) identification of hair shafts we have already discussed.

(*d*) We have been unable to confirm Haythorn's (1931) observations on the resemblance of the silver-stained basement membrane of rodent ulcers to that of the hair follicle. Indeed it would be surprising if a hair matrix tumour developed a basement membrane like the " membrana vitrea " of the root sheath. We have not examined the neurofibril content of our tumours, but Willis (1948) has indicated the unreliability of the evidence they provide.

(*e*) Haythorn (1931) describes atypical proliferations of hair follicles producing appearances which he interprets as the early stages of rodent ulcer formation. But nearly all these changes occurred in hairs surrounded by squamous carcinomata. Their occurrence is therefore rather to be taken as evidence that hair follicles react in this sort of way (it may be merely a disturbance of ordinary cyclical regeneration) to any injury, and to be a warning that apparent transitional changes in hair follicles even within a rodent ulcer may be quite meaningless.

(*f*) An argument which Haythorn (1931) does not consider is that based on the site of origin of the tumours. The hair matrix lies in most areas in the deepest part of the dermis or even deeper. A tumour arising here would spread subcutaneously. But rodent ulcers grow practically always (there was no demonstrable exception in our series) primarily between dermis and epidermis. This applies even to the epithelioma adenoides cysticum type of tumour, at least in all the cases we have seen (Fig. 1). Deep-lying skin tumours, for which a direct origin from formed adnexae must be postulated, are probably always either hidradenomata (Gates, Warren and Warvi, 1943) or the so-called benign calcifying epitheliomata (Côté, 1936), which we think is (at least in its most characteristic variety) the only true hair matrix tumour. Neither of these tumours belongs to the rodent ulcer group.

*Epidermal origin of the rodent ulcer.*

We join with the great majority of authors in finding no fault with Krompecher's (1900) original derivation of the rodent ulcer by proliferation from the under surface of the epidermis. Where we find it necessary to disagree with them is in the assumption that this necessarily involves a specifically basal-cell origin. As von Hansemann (1907) was first to point out, the surface epithelium and squamous carcinoma arise equally from the basal layer of the epidermis. It seems, however, to be generally believed that if squamous carcinoma and rodent ulcer are different tumours they must have separate cells of origin. This is not necessarily so. The epidermis has two functions: one is to supply the body with its protective coat, the other the production of adnexae. At the time of first formation of hair follicles the epidermis possesses only a rudimentary "peritrichous" outer layer, and all the capacity for growth in either of its two main directions resides in one layer of cells. Of the nature of the stimuli to these two forms of proliferation and differentiation we are largely ignorant. The stimulus to active keratin-ward proliferation (whatever its proximate mechanism) must be found in some contact with the external world—though there must be a basal level of activity of the degree seen in implantation cysts. The stimulus to the adnexa-ward proliferation almost certainly comes from some form of organizer-like action of the mesoderm. Direct evidence for this in mammals is lacking, studies of hair genesis so far available (Danneel, 1931; Trotter, 1932) being purely anatomical. But in birds, where the large size of the feather papilla makes transplantation experiments possible, the work of Lillie and his school has produced a great deal of evidence on the process of morphogenesis (Lillie, 1942), and it is known that the original stimulus to feather growth arises in the dermis (Wang, 1943), though the type of epithelium responding to the stimulus partly determines the type of feather to be formed. In the absence of evidence to the contrary it seems reasonable to assume that in mammals hairs and sweat glands are evoked from the epidermis by similar stimuli derived from the dermis.

Thus we have two stimuli, two responses, and two very different forms of tissue formed from the same layer of cells. Correspondingly we have two forms of tumour. It is simplest (though perhaps not entirely necessary) to state the resultant hypothesis in terms of Pullinger's (1949) theory of tumour origin in general. According to this, squamous carcinoma would be the result of the acquirement by epidermal cells of the ability to produce within themselves some stimulating substance which is normally only derived by external irritation, and rodent ulcer is the result of the acquirement by the same cells of the ability to produce an altogether different substance—a substance which is the normal stimulus to growth of the adnexae and which is normally derived only from the dermis.

Why then, if it results from the application of the same stimulus to the same tissue, is the rodent ulcer so poor a copy of hair follicle or sweat gland? A possible answer may lie in the importance of the connective-tissue framework in the differentiation of adnexae, a matter concerning which there is some experimental evidence. Medawar (1944) noted a phenomenon seen in rabbit skin homotransplants, which one of us has been able to study during the course of experiments reported elsewhere (Dempster, Lennox and Boag, 1950). The relevant experiments involve the transplantation of small whole thickness grafts of skin from one rabbit to another. The epidermis dies within ten days, but the dermis survives (at least as a formed structure—cellular death may occur earlier) for some weeks. In the interval the bared surface of the graft dermis is often covered by host epidermis. This new epidermis grows rapidly into the holes left in the donor dermis by the death of the donor hair follicle epithelium, and within less than four days differentiates into well-formed hair follicles (Fig. 14). The graft is thrown off before actual hairs have time to form, but differentiation is otherwise complete. The fact that surface epidermis in the adult retains this remarkable capacity to form new hairs given the appropriate stimulus seems to dispose of the necessity of assuming—as Foot (1947) did—that adnexa-like tumours can only be formed in the adult from rests of foetal material left in the epidermis.

The presence of a preformed collagen framework is then, surprisingly enough, a sufficient stimulus to the differentiation of a hair follicle. It is not unreasonable to assume that the right framework could equally evoke a sweat gland. It is possible that in the foetus the dermis supplies to the epidermis simply a non-specific stimulus to downward proliferation, and that the shape (not simply the microscopically visible shape, perhaps) of the connective-tissue matrix into which it grows determines the type of structure formed. If that were so the rodent ulcer would be easy to understand. It is a downgrowth of cells which have all the cellular potentiality for differentiation of all the skin adnexae, but which fail to make contact with the right connective-tissue matrix and so cannot produce an organized structure. Without a gland basement membrane they cannot assume the polarity and pattern of a gland, even though they can secrete ; without a hair papilla they will not form a hair shaft, even though they can keratinize.

This hypothesis is in some respects a development of that of Kyrle (1916). Though somewhat speculative, especially in its latter parts, it does at least seem capable of explaining the peculiarities of this remarkable tumour better than most of its numerous predecessors.

### SUMMARY.

1. An attempt to classify a series of 150 rodent ulcers according to the method of Foot (1947) failed.

2. Four forms of differentiation were selected for special study, chiefly because their presence or absence could be readily assessed. They were palisading (present in 73 per cent of cases), fluid formation (58 per cent), whorls (36 per cent), and melanin (35 per cent).

3. An analysis of the clinical behaviour of tumours showing each of these forms of differentiation revealed no differences between them.

4. The four forms of differentiation associated together in a purely random manner ; it is argued that this would be improbable if in fact there existed any histologically distinct varieties of the rodent ulcer.

5. Fluid formation was found to be much commoner in rodent ulcers than is generally supposed. It is suggested that it is an active secretion of the tumour cells, and may be sweat.

6. Doubt is thrown on the existence of abortive hair follicles in the tumours, and of the baso-squamous group ; both depend on doubtful interpretations of the structures here called whorls.

7. A gradation in behaviour of rodent ulcers depending on the degree but not the type of differentiation is demonstrated. The better differentiated are less common on the face, appear in younger patients, recur less frequently and respond better to irradiation. The extreme ends of this series are represented by the epithelioma adenoides cysticum, and by a group of undifferentiated tumours whose status is doubtful, but which behave like rodent ulcers.

8. A hypothesis is proposed ascribing the rodent ulcer to the autogenous production by the epidermis of a stimulus to proliferation normally supplied by the foetal dermis.

Our thanks are due to Professor J. H. Dible, Dr. C. V. Harrison, Dr. I. Doniach and Dr. A. G. E. Pearse for their help and criticism, to Dr. Constance Wood and Dr. Lilian Walter for their help with the clinical aspects, to Dr. D. A. Mitchison and Mr. J. W. Boag for their assistance with the statistics, to Dr. M. E. Lennox for the analysis of many case-histories, to Mr. E. V. Willmott for the photomicrography, and Mr. J. Griffin for the histological preparations.

### REFERENCES.

BORRMAN, R.—(1904) *Z. Krebsforsch.*, **2**, 1.
BROOKE, H. G.—(1892) *Brit. J. Derm.*, **4**, 269.
CÔTÉ, F. H.—(1936) *J. Path. Bact.*, **43**, 575.
DANNEEL, R.—(1931) *Z. Morph. Okol. Tiere*, **20**, 733.
DARIER, J., AND FERRAND, M.—(1922) *Ann. Derm. Syph., Paris*, VI ser., **3**, 385.
DEMPSTER, W. J., LENNOX, B., AND BOAG, J. W.—(1950) *Brit. J. exp. Path.*, **31**, 670.
FISHER, R. A.—(1941) ' Statistical Methods for Research Workers,' 8th ed. Edinburgh.
FOOT, N. C.—(1947) *Amer. J. Path.*, **23**, 1.
FORDYCE, J. A.—(1892) *J. cutan. Dis.*, **10**, 459.
GATES, O., WARREN, S., AND WARVI, W. N.—(1943) *Amer. J. Path.*, **19**, 591.
VON HANSEMANN, D.—(1907) *Berl. klin. Wschr.*, **44**, 723.
HAYTHORN, S. R.—(1931) *Amer. J. Cancer*, **15**, 1969.
JACOB, A.—(1827) *Dublin Hosp. Rep.*, **4**, 232.
KROMPECHER, E.—(1900) *Beitr. path. Anat.*, **28**, 1.
KYRLE, J.—(1916) *Arch. Derm. Syph., Wien*, **121**, 246.
LACASSAGNE, —(1933) *Ann. Derm. Syph., Paris*, VII ser., **4**, 497, 613 and 722.
LENNOX, B.—(1949) *J. Path. Bact.*, **61**, 587.
LILLIE, F. R.—(1942) *Biol. Rev.*, **17**, 247.
MALLORY, F. B.—(1910) *J. Amer. med. Ass.*, **55**, 1513.
MARCHIONINI, A.—(1928) *Schweiz. med. Wschr.*, **58**, 1055.
MEDAWAR, P. B.—(1944) *J. Anat.*, London, **78**, 176.
MOLESWORTH, E. H.—(1927) *Med. J. Aust.*, **1**, 878.
MONTGOMERY, H.—(1928) *Arch. Derm. Syph., N.Y.*, **18**, 50.—(1935) *Radiology*, **25**, 8.
OWEN, MAY.—(1930) *Arch. Path.*, **10**, 386.
PEMBERTON, R., CAJORI, F. A., AND CROUTER, C. Y.—(1929) *Ann. intern. Med.*, **2**, 1243.
PULLINGER, BEATRICE D.—(1949) *Lancet*, ii, 823.
REGISTRAR-GENERAL—(1949) Statistical Review of England and Wales for the year 1947, Part 1, Medical, London (H.M. Stationary Office).
RONCHESE, F.—(1933) *Amer. J. Cancer*, **18**, 875.

SEQUEIRA, J. H.—1901 *Brit. med. J.*, i, 332.
SHREK, R.—(1941*a*) *Arch. Path.*, **31**, 422.—(1941*b*) *Ibid.*, **31**, 434.
*Idem* AND GATES, O.—(1941) *Ibid.*, **31**, 411.
TELOH, H. A., AND WHEELOCK, M. C.—(1949) *Ibid.*, **48**, 447.
TROTTER, MILDRED.—(1932) In Cowdry's ' Special Cytology,' New York, vol. i, p. 41.
WALLACE, S. A., AND HALPERT, B.—(1950) *Arch. Path.*, **50**, 199.
WANG, H.—(1943) *Physiol. Zool.*, **16**, 325.
WARREN, S., GATES, O., AND BUTTERFIELD, P. W.—(1936) *New Engl. J. Med.*, **215**, 1060.
WILLIS, R. A.—(1945) *Cancer Res.*, **5**, 469.—(1948) ' Pathology of Tumours,' London (Butterworth).